# Not Just a Pathogen? Description of a Plant-Beneficial *Pseudomonas syringae* Strain

**DOI:** 10.3389/fmicb.2019.01409

**Published:** 2019-06-21

**Authors:** Alessandro Passera, Stéphane Compant, Paola Casati, Maria Giovanna Maturo, Giovanna Battelli, Fabio Quaglino, Livio Antonielli, Domenico Salerno, Milena Brasca, Silvia Laura Toffolatti, Francesco Mantegazza, Massimo Delledonne, Birgit Mitter

**Affiliations:** ^1^Department of Agricultural and Environmental Sciences – Production, Landscape, Agroenergy, Università degli Studi di Milano, Milan, Italy; ^2^Center for Health & Bioresources, AIT Austrian Institute of Technology GmbH, Tulln, Austria; ^3^Functional Genomics Laboratory, Department of Biotechnology, University of Verona, Verona, Italy; ^4^Institute of Sciences of Food Production, Italian National Research Council, Milan, Italy; ^5^Department Medicina e Chirurgia, Università degli Studi di Milano-Bicocca, Vedano al Lambro, Italy

**Keywords:** *Pseudomonas syringae*, biocontrol, pangenome analysis, confocal microscopy, *Botrytis cinerea*

## Abstract

Plants develop in a microbe-rich environment and must interact with a plethora of microorganisms, both pathogenic and beneficial. Indeed, such is the case of *Pseudomonas*, and its model organisms *P. fluorescens* and *P. syringae*, a bacterial genus that has received particular attention because of its beneficial effect on plants and its pathogenic strains. The present study aims to compare plant-beneficial and pathogenic strains belonging to the *P. syringae* species to get new insights into the distinction between the two types of plant–microbe interactions. In assays carried out under greenhouse conditions, *P. syringae* pv. *syringae* strain 260-02 was shown to promote plant-growth and to exert biocontrol of *P. syringae* pv. *tomato* strain DC3000, against the *Botrytis cinerea* fungus and the *Cymbidium Ringspot Virus*. This *P. syringae* strain also had a distinct volatile emission profile, as well as a different plant-colonization pattern, visualized by confocal microscopy and *gfp* labeled strains, compared to strain DC3000. Despite the different behavior, the *P. syringae* strain 260-02 showed great similarity to pathogenic strains at a genomic level. However, genome analyses highlighted a few differences that form the basis for the following hypotheses regarding strain 260-02. *P. syringae* strain 260-02: (i) possesses non-functional virulence genes, like the mangotoxin-producing operon *Mbo*; (ii) has different regulation pathways, suggested by the difference in the autoinducer system and the lack of a virulence activator gene; (iii) has genes encoding DNA methylases different from those found in other *P. syringae* strains, suggested by the presence of horizontal-gene-transfer-obtained methylases that could affect gene expression.

## Introduction

Since the beginning of their evolution, plants have interacted with microorganisms. Such interaction could be with beneficial organisms, helping the plants to colonize land (Vandenkoornhuyse et al., 2015) and aiding their development and health, or with pathogens, which undermine plant health.

These interactions are important determinants in plant life and are extremely complex, making it difficult to understand them in their entirety. While several pathways and plant–microbe interactions have been described and are understood, little is known about how they fit into the overall picture of plants, where beneficial organisms and pathogens interact in a complex scenario that involves entire microbial communities and environmental factors (Rosier et al., 2018).

Research into these interactions has demonstrated that both beneficial and pathogenic microorganisms employ very similar, if not identical, strategies when they come into contact with a plant host; indeed, they face the same problem: how to establish themselves and survive in a plant? Thus, the microorganisms must avoid detection by the plant or be able to dampen the plant defense response, and they need to find a way to obtain nutrients from the host (Brader et al., 2017).

To give an example: a very widespread mechanism employed by plant-beneficial bacteria in the host is to reduce the level of ethylene, a stress hormone. This occurs by diverse mechanisms e.g., direct ethylene degradation or deamination of the precursor molecule, 1-aminocyclopropane-1-carboxylate (Wang et al., 2000). While this mechanism does indeed promote plant growth by preventing the inhibitory effect otherwise caused by ethylene on plant growth, the same mechanism is also employed by pathogenic bacteria as ethylene is a key signal molecule in the activation of defense reactions; therefore, a plant with low ethylene levels is often more susceptible to infection by necrotrophic pathogens (Nascimento et al., 2018).

The fact that many plant–host interaction pathways are shared by plant-beneficial and pathogenic organisms highlights the problem of successfully managing to discriminate between the two: whereas in the past it was believed that some traits were characteristic of one kind of microorganism or the other, the distinction is becoming more blurred as knowledge of the base mechanisms behind plant–microorganism interaction becomes clearer. This difficulty in discriminating between beneficial and pathogenic organisms has a very practical impact on agriculture as current international policies promote sustainable methods for the management of crop cultivation, biological fertilizers and pesticides. Despite this support, the use of microorganism-based products faces limitations, given the variable and unpredictable results, when employed in different conditions and on different hosts. This means that there is an urgent need for faster methods to ascertain the potential and safety of a bacterial isolate for use in agriculture. Furthermore, more general conclusions need to be drawn so that agriculture, as a whole, can be helped, rather than just concentrating on the new data available for specific host plants and bacterial strains (Rosier et al., 2018).

In the scenario of plant–bacteria interaction, one of the most investigated genera is surely *Pseudomonas*, a genus of Gram-negative, rod-shaped bacteria that includes several plant-beneficial species (Pieterse et al., 2014), as well as important pathogenic species, such as *Pseudomonas syringae*, which is studied as a model to elucidate several key plant–pathogen interactions (Dangl and Jones, 2001; Jones and Dangl, 2006).

While several strains of *P. fluorescens*, *P. putida*, and *P. protegens* are known to have beneficial effects on different plants, *P. syringae* is a highly pathogenic species that contains different subgroups specialized for different plants, from grasses to arboreal plants, giving the species, as a whole, an impressive host range (Baltrus et al., 2017). The study of *P. syringae*, a model organism for plant–pathogen interaction, led to the discovery of the mechanisms through which it colonizes and affects the host. Of particular relevance for *P. syringae* is its vast range of effectors, which are directly injected into plant cells through the type III secretion system, subverting several cellular functions and favoring the establishment of pathogenesis (Macho et al., 2012).

Several studies were carried out to compare the phenotype and genome of pathogenic *Pseudomonas*, particularly the plant pathogen *P. syringae* and the human pathogen *P. aeruginosa*, with non-pathogenic strains of related bacteria, such as *P. putida* or *P. fluorescens*. The main objective of these studies was to gain insight into the differences between these isolates and, therefore, into the determinants of the pathogenic or beneficial behavior (Paulsen et al., 2005; Lindeberg et al., 2008; Shen et al., 2013).

The current study characterizes an isolate of *P. syringae*, called 260-02, identifying it as a plant-beneficial strain. It compares the isolate with a well-known pathogenic strain, *P. syringae* DC3000, to assess their differences in phenotypic behavior in *in vitro* and *in planta* conditions e.g., their ability (i) to inhibit the growth of the plant pathogenic fungus *Botrytis cinerea*, (ii) to produce volatile compounds, (iii) to colonize tissues and cause symptoms in pepper and tomato plants. The study also investigated strain 260-02 at the genomic level, comparing it to several pathogenic *P. syringae* strains in order to identify genetic elements that could be related to its beneficial behavior, despite a great portion of its genetic background being shared with pathogenic strains.

## Materials and Methods

### Bacteria Isolation, Transformation and Cultivation

In May 2012, as part of a survey on the apple proliferation disease caused by “*Candidatus* Phytoplasma mali,” leaves were taken from apple trees (*Malus pumila* Miller) at the Minoprio Foundation (Como, Italy) and endophytic bacteria were isolated from this material. Among the isolated strains was *P. syringae* pv. *syringae* strain 260-02. The plant showed no symptoms of the disease, and the pathogen was not detected by specific nested-PCR assays.

Instead*, P. syringae* pv. *tomato* strain DC3000 was obtained from the collection of the University of Natural Resources and Life Sciences of Vienna (BOKU, Austria).

Both strains were cultivated on LB High Salt Agar plates (tryptone 10 g/L, yeast extract 5 g/L, sodium chloride 10 g/L, agar 15 g/L) at 25°C, and were stored in a 20% glycerol solution at –80°C for long conservation periods.

The strains were transformed with the integrative plasmid PUT*gfp*2× (Tombolini et al., 1997), carrying, as markers, the genes encoding for kanamycin resistance and the production of Green Fluorescent Protein. Several transformed colonies for strain DC3000 were compared to the wild-type to determine which ones had the most similar growth speed and behavior. The result of this protocol was the *gfp*-labeled strain, referred to in the manuscript as DC3000::*gfp*.

Since strain 260-02 did not express visible fluorescence after transformation with this method, it was transformed with the replicative plasmid pHM2-GFP (Favia et al., 2007). The result of this protocol was the *gfp*-labeled strain, referred in the manuscript as 260-02:*gfp*.

### Fungi Isolation and Cultivation

Strain MG53 of *B. cinerea* Pers. (BC) is used in fungal pathogen antagonism assays, the strain having been first isolated from wheat kernel in 2014. The strain was conserved in the fungal culture collection of the Mycology Laboratory at the Department of Agricultural and Environmental Sciences (DiSAA), University of Milan, Italy. The isolate was cultivated on potato dextrose agar (PDA, Difco^TM^) at 20°C and stored at 4°C.

### Genome Sequencing and Assembly

Genomic DNA was isolated from strain 260-02 as follows: the strain was cultivated in LB broth at 25°C overnight and the genomic DNA was extracted using GenElute^TM^ Bacterial Genomic DNA Kit (Sigma-Aldich), following the manufacturer’s instruction. Genomic DNA was quantified with the Qubit dsDNA HS Assay kit (Life Technologies), purity and integrity were assessed with Nanodrop 1000 spectrophotometer (Thermo Fisher Scientific) and by agarose gel electrophoresis, respectively.

Illumina libraries were produced starting from 1 μg of genomic DNA, which was sheared using the Covaris S220 instrument (Covaris Inc., Woburn, MA). Size selection of fragments 500 bp in length was conducted on agarose gel at 1.8% and libraries were produced using TruSeq DNA Sample Prep Kit (Illumina, San Diego, CA) according to manufacturer instructions. Sequencing was performed on a HiSeq1000 instrument with 100 × 2nt Pair end protocol using the TruSeq PE Cluster v3 kit (Illumina, San Diego, CA) according to manufacturer instructions.

Illumina reads underwent a quality filtering process before being assembled *de novo* as follows: (1) low quality reads with more than 10% of undetermined bases (Ns) or with more than 50 bases called with a phred-scored basecall quality < 7 were filtered out; (2) reads were then de-duplicated using a custom in-house script; (3) sequencing adaptors were clipped with Scythe (version 0.994) using default parameters and low quality 3′ ends of reads were quality trimmed with Sickle using a quality threshold of 20 over a window of 10 bases and discarding reads under 25nt in length.

Filtered reads were quality corrected with BayesHammer (Nikolenko et al., 2012) and then assembled using SPAdes 2.9.0 (Bankevich et al., 2012). Assembly was performed with multiple k-mer combinations in the 75 to 97 range, the best assembly occurring with k-mers 79, 83, 89, 91, 93: these showed the least fragmented sequences, the least number of contigs with the highest N50, mean and median scaffold lengths. The assembled sequences were investigated for putative assembled plasmid genomes by BLAST search, against the NCBI plasmid genomes database.

Gene annotation was performed using the RAST web service, and the functional annotation of protein coding genes was improved with Blast2GO (ver. 2.8) (Conesa et al., 2005).

Gene clusters related to the production of secondary metabolites was predicted using the antiSMASH 3.0 online tool (Weber et al., 2015).

### Phylogenesis

Although running a BLAST of the full-length 16S rRNA gene sequence from the genome of 260-02 was sufficient to characterize the strain as *P. syringae*, more in-depth phylogenetic analyses were carried out. To discern the pathovar more similar to strain 260-02 a phylogenetic tree was computed, using a concatenated nucleotide sequence of the genes *rpoD*/*gyrB* (Yamamoto et al., 2000) obtained from several complete genomes of bacteria from the *Pseudomonas* genus (Supplementary Table S1). The species was also confirmed using the 40-markers approach of the software Phylosift (Darling et al., 2014), and the average nucleotide identity among the full genomes of *P. syringae* (Supplementary Table S1) was compared through ANI-MUMmer.

The mangotoxin operon, a feature shared by strain 260-02, and its closest relative from the database, the strain UMAF0158, was also compared using a phylogenetic approach to determine similarity on the basis of a known pathogenesis-related function, focusing particularly on the amino acid sequence encoded by the gene *mboE* (Supplementary Table S1).

All the phylogenetic trees were obtained by aligning the sequences through the ClustalW algorithm, manually controlling the resulting alignments, and compiling the trees using MegaX software (Kumar et al., 2018).

### Dual-Culture Inhibition

*Botrytis cinerea* growth inhibition by the two bacterial strains was assessed through the dual-culture plate method described in Passera et al. (2017). Briefly: droplets from an overnight liquid culture of either strain 260-02 or strain DC3000 (approximately 2 × 10^6^ CFUs) were placed on four cellulose disks around the inside edge of a Petri dish containing a Tryptone Glucose Yeast Extract Agar medium (TGYA) – 5 g/L tryptone, 1 g/L glucose, 3 g/L yeast extract, 15 g/L agar. After 2 days of incubation, a BC plug (0.5 cm in diameter) was taken from actively growing mycelium of BC and was placed in the middle of the plate. As negative controls, plates containing (i) BC alone, (ii) BC with blank sterilized filter paper discs, and (iii) BC and discs inoculated with 20 μl of sterilized LB broth were used.

Fungal growth, as mycelial growth diameter, was measured 5, 7, and 14 days post-inoculation (dpi). Each test was carried out with plates in triplicate and three independent measures were made for each plate at each measuring time. The Growth Inhibition Percentage (GIP) was calculated as [1–(D1/D2)] × 100, where D1 is the radial colony growth on the bacteria-treated plate, D2 is the radial colony growth on the control plate.

### Dual-Plate Inhibition

To evaluate strains 260-02 and DC3000’s ability to inhibit fungal growth through the production of volatile compounds, a dual-plate assay was carried out (Chaurasia et al., 2004). Briefly, 100 μl of an overnight strain of both the 260-02 and DC3000 cultures, in LB broth (approximately 10^7^ CFU), were diffused on the surface of a TGYA plate and incubated at 25°C. After 2 days, a fungal mycelial plug (0.5 cm in diameter) was taken from actively growing mycelium of BC and was inoculated onto another TGYA plate. Under sterile conditions, the lid of the plate bearing the bacteria was replaced by the upturned plate containing the fungal inoculum, and the plates were sealed together with Parafilm. After fungal inoculation, all the plates were kept at 25°C in the dark, and the fungal growth was measured 14 dpi. Each test was made with plates in triplicate, three independent measures being made for each plate. The growth inhibition percentage, determined by volatile compounds (GIPv), was calculated as previously described.

### Production of Volatiles

An analysis of the VOCs produced by strains 260-02 and DC3000 was carried out using a method that reproduces the dual-plate assay on the inside of a 20 ml headspace vial. Inside the vial, two layers of TGYA (described as before, except using 3% agar) were prepared on opposite sides of the vial, making sure that the layers did not touch one another. This permitted the inoculation of the different microorganisms on one layer, with no contact with the other. The vials were then sealed with a cap containing a membrane through which the extraction fiber was inserted, without coming into contact with the solid media or the microorganisms, only with the volatile compounds present in the space between the two. To test which volatiles were produced by the bacterial strain and by the fungal strain, the vials were prepared thus: (i) vials in which only strain 260-02, or strain DC3000, was growing, (ii) plates on which only BC was growing, (iii) vials in which both strain 260-02 and BC were growing, and (iv) vials with only TGYA medium, to be used as negative controls. The inoculation of both fungus and bacteria was carried out in quick succession, to avoid losing VOCs by opening the vial before the analysis. The vials, prepared in triplicates, were then closed with their cap and sealed with parafilm, and left in incubation at 25°C in the dark for 14 days before VOC analysis. This was performed using the Solid-Phase-Micro-Extraction technique, followed by Gas Chromatography-Mass Spectrometry (SPME-GC-MS). The VOCs were adsorbed on a Divinylbenzene/Carboxen/Polydimethylsiloxane (DVB/ CAR/PDMS) fiber and desorbed at 260°C in the injection port of an Agilent Technologies 6890N/5973N gas chromatograph-mass spectrometer equipped with a 60 m × 0.25 mm × 0.25 μm 100% polyethylene glycol column (Zebron ZB-WAX plus, Phenomenex). The carrier gas was helium, used in constant pressure mode (150 kPa). The oven was programmed at 45°C (5-min hold) and ramped up to 219°C at 6°C/min intervals (16-min hold). The transfer line to the mass spectrometer was maintained at 280°C, while the ion source was 230°C, and the quadrupole was 150°C. Acquisition was performed in electronic impact mode. VOCs were identified using the Wiley 7n-1 MS library on Agilent MSD ChemStation software (Agilent Technologies Inc.). Confirmation of the identity of the volatile compounds was achieved by comparing the GC retention indices and mass spectra of individual components with those of authentic reference compounds (Sigma-Aldrich Co.) under the same operating conditions. The data collected refer to the peak area of the quant ion of each compound, and are expressed as a natural logarithm, for this reason the determination must be considered semi-quantitative. Compounds were considered “absent” (i.e., not produced) when the signal to noise ratio was below 2:1. To check the presence of carry-over effects, blank extractions were conducted regularly.

### Inhibition by Filtrate

To evaluate strains 260-02 and DC3000’s ability to inhibit fungal growth through non-volatile compounds secreted in the growth medium, an assay was carried out: TGYA plates containing the strain’s culture broth, previously purified of all cells, were prepared and used as substrate for the growth of the fungi, as described by Passera et al. (2017). After 72 h of incubation, the liquid culture was purified of bacterial cells by centrifugation (6800 × *g* for 10 min) and filtration (0.2 μm pore size). The filtrate, at a final concentration of 50% v/v, was added to the TGYA medium to prepare the plates. To control the plate sterility, the plates were incubated at 25°C overnight and checked for bacterial growth. After preparing the plates with the culture filtrate, BC was inoculated under sterile conditions. The inoculum was a 0.5 cm diameter mycelial plug excised with a sterilized cork borer from the edges of an actively growing fungal culture. It was placed in the middle of the TGYA plates supplemented with filtrate. As negative control, TGYA plates, supplemented with sterile LB broth, were used for fungal culture. After fungal inoculation, all the plates were incubated at 25°C in the dark. Fungal growth was measured 14 dpi as mycelial growth diameter. Each test was carried out with plates in triplicate and three independent measures were made for each plate. Growth inhibition percentage from cultural filtrate (GIPf) was calculated as described previously.

### Biocontrol Against *B. cinere*a

The ability of strains 260-02 and DC3000 to reduce infection from BC was evaluated in an experiment carried out on detached tomatoes in postharvest conditions. For this assay, ripe and healthy cherry tomatoes, grown organically in Italy and purchased in a local grocery, were used. Detached tomatoes of uniform size, free of visible blemishes, were surface sterilized in a 70% ethanol solution in water for 5 min, rinsed in water three times, and dried on filter paper under a laminar flow hood. After drying, the equatorial area of each was pierced 4 times by a needle. Bacterial strains 260-02 and DC3000 were inoculated singly by a 5 min soaking of each tomato in a bacterial suspension (approximately 10^6^ CFUs/ml in Ringers solution, Sigma-Aldrich), and then left to dry on filter paper under a laminar flow hood. The BC conidia were inoculated by applying a 20 μl drop of conidial suspension (5 × 10^5^ conidia/ml) into each puncture wound. For each treatment (non-treated, bacterial strains 260-02 or DC3000 alone, BC alone, bacterial strain 260-02 or DC3000 and BC) 10 tomatoes were put on a sterile ceramic tray in a glass chamber, containing a wet piece of filter paper to maintain a relative humidity of 95% inside the chamber, and incubated at 20°C in the dark. All the aforementioned procedures were carried out under sterile conditions. Each treatment was carried out in triplicate. The tomatoes were evaluated visually to determine fungal colonization at 7 days after inoculation.

The results were expressed as visual classes, according to the scale presented in a previous work (Vercesi et al., 2014). Visual classes were transformed into a percentage infection index (I%I) according to the formula proposed by Townsend and Heuberger (1943).

### Plant-Growth Promotion

The ability of strain 260-02 to promote growth of *Solanum lycopersicum* L. var. Sibari F1 (referred to as “tomato” in the rest of the study), and *Capsicum annuum* L. var Zebo F1 (referred to as “pepper” in the rest of the study) plants was assayed under greenhouse conditions. Fifteen days-old seedlings of both plant species were inoculated by root dipping with a PBS solution containing 10^5^ CFU/ml of strain 260-02 or mock-inoculated using only PBS. The height of these plants, 7 per species per treatment, was monitored weekly over a period of 2 months and compared between treatments. The plants inoculated in this way were also used for biocontrol trials against different pathogens, described below.

### Biocontrol Against *P. syringae* pv. *tomato* DC3000

The tomato and pepper plants, either mock-treated or inoculated with strain 260-02, were inoculated by spraying a PBS cell suspension of the pathogen (10^5^ CFU/ml) onto the plants leaves 2 weeks after the root-dip inoculation of bacteria. For each condition, 7 biological replicates were set up. Symptom development was monitored one and 2 weeks after inoculation with the pathogen, evaluated as the number of necrotic spots visible on the leaf surface, and compared with the non-treated control.

### Biocontrol Against *Cymbidium Ring Spot Viru*s

The pepper plants, either mock-treated or inoculated with strain 260-02, were mechanically inoculated with *Cymbidium Ring Spot Virus* (CymRSV) 1 month after the root-dip inoculation of bacteria. Inoculum of the virus was obtained by grinding leaves of infected *Nicotiana benthamiana* plants in a 0.05 M phosphate buffer (pH 7, containing DIECA at 5 mM and EDTA at 1 mM). For each condition, 7 biological replicates were set up. Severity of systemic infection (I%S) was evaluated as a percentage defined as (SL/TL)^*^100, where SL is the number of symptomatic leaves that were not mechanically inoculated, and TL is the total number of leaves that were not mechanically inoculated.

### Colonization Assays

The ability of the analyzed strains to colonize plants from two different tissues that are important interfaces between plant and environmental microorganisms, root and leaf, was tested *in planta* using two Solanaceae plant crops: tomato and pepper. Seven-day old seedlings were inoculated by root dipping with a PBS solution containing 10^5^ CFU/ml of either strain 260-02:*gfp* or of DC3000::*gfp* to test the strains’ ability to colonize the roots.

To describe the ability to colonize the leaves and induce symptoms of the strains, either wild-type or transformed, tomato and pepper plants fifteen-days old were sprayed with a PBS solution containing 10^5^ CFU/ml of either strain 260-02, 260-02:*gfp*, DC3000, or DC3000::*gfp*. As part of this assay, in order to visualize the biocontrol effect of the root inoculation of strain 260-02 against the establishment of DC3000 on leaves, and to clarify whether it was a strain-specific response or a general plant response to exposure to *P. syringae*, additional plants were sprayed with DC3000::*gfp* on the leaves. These plants had been root-dipped in either a solution of strain DC3000 or strain 260-02 (as described above) 1 week before the foliar inoculation of the pathogen.

For both root and leaf experiments, a mock-treated control inoculated with PBS containing no bacterial inoculum was performed. The treatments were carried out in five replicates for tomato plants, and three replicates for pepper plants. Both root and leaf samples were examined with a confocal microscope (Olympus FluoView FV1000 with multiline laser FV5-LAMAR-2 HeNe(G) and laser FV10-LAHEG230-2) after 3 and 6 days of inoculum. For the acquisition of GFP fluorescence, the filter settings were 488 nm excitation, 515 nm emission, while for the acquisition of autofluorescence from plant molecules (chlorophyll, phenolic compounds) (Garcia-Plazaola et al., 2015) the filter settings were 594 nm excitation, 637 nm emission. For each treatment, five samples, either whole leaves or whole roots cleaned from soil, were excised from the plant with a scalpel, directly put on a glass slide and covered with a few drops of sterile PBS, with no further preparation. Absence of fluorescence ascribable to GFP in mock samples was evaluated as well to exclude the possibility of false positives. Furthermore, for leaf inoculation, development of symptoms was visually evaluated 3 and 6 days after inoculum.

Each whole sample, either leaf or root, was visually examined at the microscope with epifluorescent light, in order to identify the portions of the sample that were suitable for capturing informative confocal microscopy pictures.

The pictures taken from the abaxial surface of leaves of pepper and tomato plants inoculated with strain DC3000::*gfp*, either treated with bacteria at the root or mock treated, were used for a quantitative analysis of bacterial presence on leaves.

A full three-dimensional scan of different views was taken, collecting 50 to 100 images at different focal planes, spaced by the focal width in height, the number of frames was chosen to include all the leaf thicknesses in the recorded volume. After acquiring the three-dimensional fluorescence data the bidimensional maximum intensity projection (MIP) was reconstructed using the following simple algorithm, first proposed in medical imaging (Wallis et al., 1989). Starting from the *x*-*y*-*z* intensity map, the maximum value of the *z* dependent intensity function was chosen for each *x*-*y* coordinate. For each MIP, the green pixels above the threshold, taken to be three times the average green intensity value, were counted and converted to an estimate of the surface colonization by bacteria (μm^2^), using FIJI (Schindelin et al., 2012) and Matlab 2018 (Mathworks Inc.) softwares. These values were then compared considering the three different treatments, based on the quantification of seven images per treatment.

### Comparative Pangenome Analysis

After having determined from the phenotype that strain 260-02 has a plant-beneficial phenotype, a comparative analysis at the pangenome level was carried out to compare the genome of the genomic level that could explain this difference in phenotype.

For this analysis, the genomes of a list of *P. syringae* strains, downloaded from NCBI (Supplementary Table S1), and the genome of strain 260-02 were annotated with PROKKA software (Seemann, 2014).

After this step, performed for a homogeneous genome annotation, the genomes were compared using the software Roary (Page et al., 2015). The main output of the software was a gene presence/absence table, which was manually examined to search for features that could be related to the different phenotypes of the bacterial strains.

### Statistical Analysis

All data comparisons made in this study were verified with statistical analyses in order to ascertain the significance of the results. All statistical tests were carried out using the IBM SPSS v. 24 statistic package. Details of how each set of data was evaluated are reported in the caption of the figures illustrating the corresponding results.

## Results and Discussion

### Genomic Overview and Features

Total nucleic acids were extracted from a pure culture of strain 260-02 and utilized to sequence its genome on an Illumina HiSeq 1000 sequencer. The assembly of the Illumina reads obtained from the sequencing of strain 260-02 yielded a draft genome divided in 43 contigs, for a total length of approximately 6.058 Mbp. The genome was deposited in the GenBank repository under Accession Number MLFM00000000, and has an overall percentage of G+C content of 59.2%; annotation with RAST found that it encodes for 5,214 genes, and 63 catalytic RNA molecules. The annotation also identified 2,658 genes encoding for proteins involved with known metabolic functions (2,512 non-hypothetical proteins, 146 hypothetical proteins), and 2,576 genes encoding for proteins with no known function (1,530 non-hypothetical proteins, 1,046 hypothetical proteins).

The annotated genome of strain 260-02 was investigated for features that could be related to plant-growth promotion and biocontrol effects, along with traits generally useful for plant-associated microbes, such as those employed to overcome plant defense mechanisms. Table 1 shows the main categories that were considered, as well as the number of genes per category.

**TABLE 1:** List of genes related to plant association, plant growth promotion, and biocontrol identified in the genome of *Pseudomonas syringae* strain 260-02.

**Function**	**No. of genes**
Biocontrol	7
Chemotaxis and motility	73
Detoxification	35
Plant growth-promoting properties	8
Quorum sensing	15
Secretion systems	108
* Type I secretion system*	1
* Type II secretion system*	11
* Type II/IV secretion system*	6
* Type III secretion system*	30
* Type IV secretion system*	23
* Type V secretion system*	6
* Type VI secretion system*	30
* Type VII secretion system*	1
Siderophores	37
Signal transduction – two-component systems	16
Stress-related enzymes	20
Transcriptional regulators	3
Transport system	50

*In normal text are reported the main categories, and when appropriate, the subcategories are indicated in italic.*

Strain 260-02 carries genes that can be related to a good fitness as a plant colonizer, carrying several genes that survive against the toxic compounds present inside plant tissues, such as catalase, super oxide dismutase, and glutathione S-transferase, as well as a high number of mono- and dioxygenases that can detoxify several compounds, including secondary defense compounds produced by plants.

The strain’s genome also possesses several chemotaxis and motility genes, indicating the ability to move in soil, on plant surfaces, and possibly reach specific zones where it can interact with a plant host.

The automatic annotation with RAST detected only a few transcriptional regulator genes, but among these is the one encoding for a regulatory protein of response to nitric oxide, a toxic compound that can be produced by plants.

The genome of the strain showed a wide array of two-component transduction systems, even if the only gene among them that had a clear function associated to it is one encoding for a type IV fimbriae expression protein PilR. On the contrary, the strain does not seem to play any active part in quorum sensing: while the strain encodes for several LuxR proteins, part of the autoinducer quorum sensing mechanism typical of Gram- bacteria (Antunes and Ferreira, 2009), there is no LuxI to go along with them, instead it encodes for a quorum quenching protein that removes the acyl group from acyl homoserine lactones, indicating a possible negative effect of the strain on quorum sensing by other bacteria (Uroz et al., 2009).

The strain’s genome also shows a very active molecular trafficking, with many transporter genes, mostly belonging to the ABC transporter family, and several bacterial secretion systems. The genome also contains a few genes related to secretion systems I, V, and VII, and several genes related to secretion systems II, III, IV, and VI, with some genes that are attributed to both secretion systems II and IV, which have some elements in common. Of these secretion systems, III, IV, and VI are thought to be the ones more deeply involved with bacteria–plant host interaction (Mitter et al., 2013) and are also the ones more abundant in the genome of strain 260-02.

Genes putatively involved directly in plant-growth promotion were observed, such as those that encode proteins of the auxin production pathway and a 1-aminocyclopropane-1-carboxylate (ACC) deaminase, which reduces the levels of ethylene in plants by degrading the precursor of this hormone, thus reducing the perception of stress in the plant host and promoting growth (Sessitsch et al., 2005).

Also, genes related to siderophores can be involved in plant-beneficial functions: siderophores can either take part in the biocontrol process, sequestering iron from pathogens, or directly contribute to the iron uptake from the plant (Pii et al., 2015). In particular, it was possible to detect several genes related to the assembly and utilization of pyoverdine, a typical siderophore of *Pseudomonas*, and achromobactin.

Other genes identified in the genome that could be directly involved in determining a biocontrol activity of strain 260-02 include those related to the production of toxic compounds, namely antibiotics such as bacteriocins and phenazine. This last compound has been characterized for its relevance in the biocontrol efficacy of several *Pseudomonas* strains (Ma et al., 2016).

### Phylogeny Analyses

**FIGURE 1:**
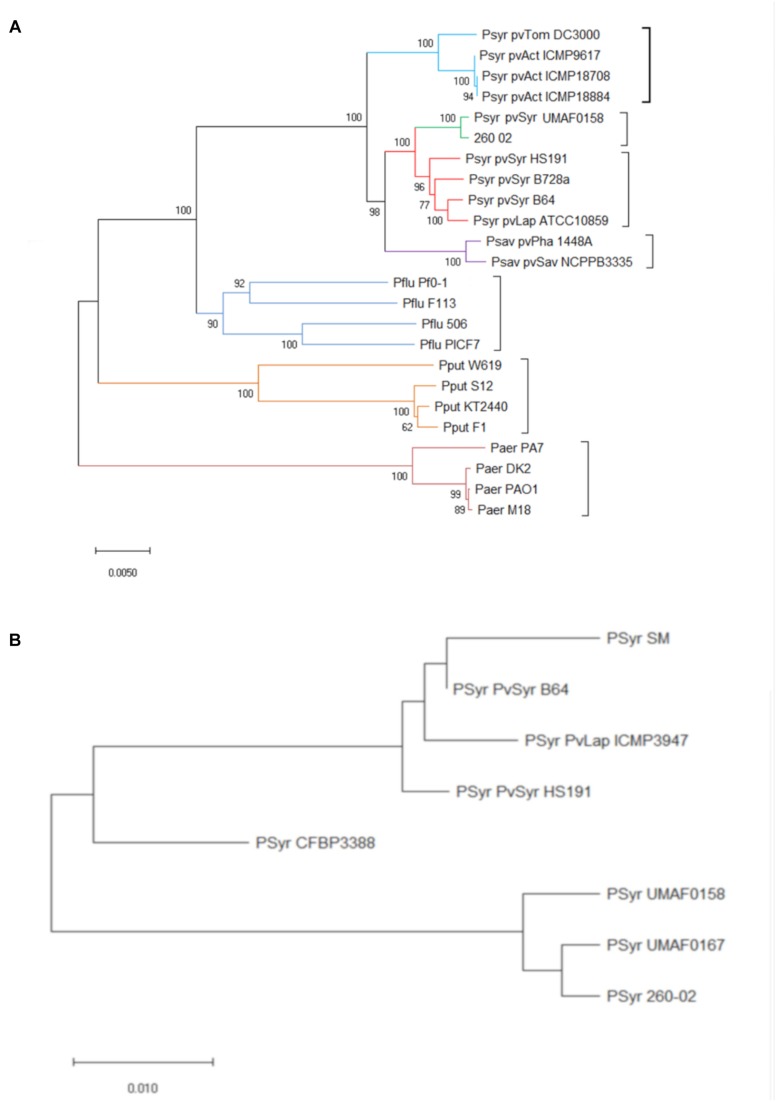
Phylogenetic positions of strain 260-02 in relation to other *P. syringae* strains. Organisms included in the analysis are reported as follows: Psyr for *Pseudomonas syringae*; Psav for *Pseudomonas savastanoi*; Pflu for *Pseudomonas fluorescens*; Paer for *Pseudomonas aeruginosa*; Pput for *Pseudomonas putida*. Furthermore, for *P. syringae* and *P. savastanoi*, pathovars are defined as follows: pvTom for pathovar *tomato*; pvSyr for pathovar *syringae*; pvAct for pathovar *actinidiae*; pvLap for pathovar *lapsa*; pvPha for pathovar *phaseolicola*; pvSav for pathovar *savastanoi* **(A)** Unrooted phylogenetic tree inferred from the *gyrB*/*rpoD* concatenated nucleotide sequence of strain 260-02 and the sequences obtained from GenBank (Supplementary Table S1); minimum evolution method was carried out using the Jukes-Cantor model and bootstrap replicated 1,000 times. Names of strains are reported on the panel **(B)** Unrooted phylogenetic tree inferred from the predicted protein sequences coded by the *mboE* gene of strain 260-02 and the sequences obtained from GenBank (Supplementary Table S1); The evolutionary history was inferred using the Minimum Evolution method. The tree is drawn to scale, with branch lengths in the same units as those of the evolutionary distances used to infer the phylogenetic tree. The evolutionary distances were computed using the Poisson correction method and are in the units of the number of amino acid substitutions per site. The tree was bootstrap replicated 1,000 times.

The genomic data obtained about strain 260-02 were used in different approaches to determine the exact taxonomy of the strain and its relation to closely related organisms. The first level of phylogenetic analysis involved constructing a concatenamer of the sequences of two highly conserved genes *rpoD* and *gyrB*, and analyzing them together by constructing a phylogenetic tree. This phylogenetic tree (Figure 1A) clearly distinguished the different species of *Pseudomonas*, inserted as outgroups, and managed to differentiate between pathovars. The branch containing strain 260-02 and its closest relative, strain UMAF0158, is part of the *P. syringae* pv. *syringae* group, but strain UMAF0158 was originally described as a pathogen of mango, a woody plant, and reported as being able to cause symptoms on tomato as well (Carrion et al., 2012). This different phylogenetic branch can be related to host adaptation. It is reported that *P. syringae* pv. *syringae* strains that colonize perennial, woody plants, such as mango and apple for strains UMAF0158 and 260-02, have some genetic differences compared to their relatives that live in herbaceous hosts (Baltrus et al., 2017). This phylogenetic attribution to the *P. syringae* pv. *syringae* was confirmed by the analysis carried out in Phylosift, and the comparison carried out with ANI-MUMmer showed that strain UMAF0158 is the closest relative of strain 260-02 with an average sequence identity of approximately 99%, while the model pathogen used in for the experimental trials, strain DC3000 has 87% of average sequence identity with strain 260-02.

Since a main component of the pathogenic effect of strain UMAF0158 is thought to be the production of mangotoxins (Arrebola et al., 2009), the sequences of the genes forming the operon encoding for the production of these toxins were likewise compared. Interestingly, the operon from strain 260-02 clusters with that of strain UMAF0167, which does not produce mangotoxins (Carrion et al., 2012), rather than with that of strain UMAF0158, which produces them. The difference between the operons was found to be localized in one gene, *mboE* (Figure 1B). While this gene encodes for an arginine deiminase, and not for the toxin itself, it has already been reported that disruption in any of the genes of this operon leads to a lack of mangotoxin production (Carrion et al., 2014). This difference alone is not sufficient to explain the completely different behavior between the beneficial strain 260-02 and pathogens, but it might be an indication that strain 260-02 could be a “disarmed pathogen” (Reinhold-Hurek and Hurek, 2011).

### *In vitro* Fungal Inhibition

**FIGURE 2:**
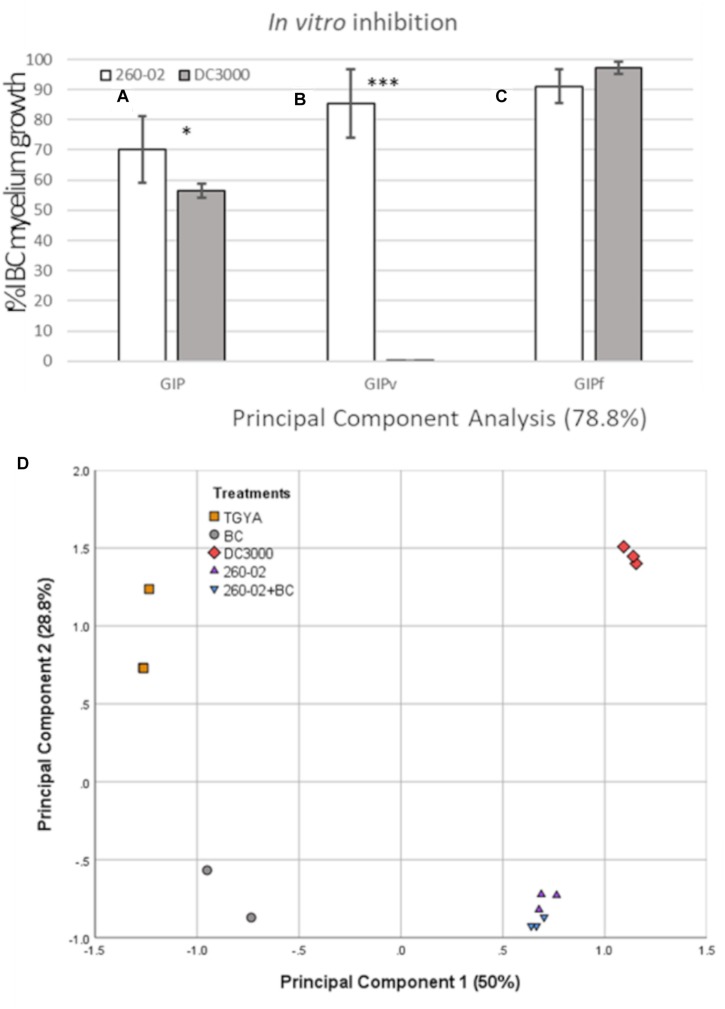
Results of the *in vitro* assays. Graph reporting on the *Y* axis the percentage of inhibition of *Botrytis cinerea* growth observed in panel **(A)** the dual-culture assay (GIP), **(B)** the dual-plate assay, measuring the effect of volatiles alone (GIPv), and **(C)** on plates containing cultural filtrate (GIPf) values registered in the competition assays against *B. cinerea* MG53 using the strains 260-02 and DC3000. The bars represent the mean value of 3 independent measures taken on 3 biological replicates of the experiment. Statistically different results according to Student’s *T*-test are reported with asterisks (^*^p < 0.05; ^∗∗^*p* < 0.01; ^∗∗∗^*p* ≤ 0.001) **(D)** Principal component analysis describing 78.8% of the difference between the volatile molecules found in the sterile medium (TGYA), those produced by BC, by strain DC3000, by strain 260-02 alone, and by strain 260-02 cultured together with BC (260-02+BC).

Strain 260-02 and the plant-pathogenic strain DC3000 were compared for their ability to inhibit the growth of a strain of the fungal pathogen *B. cinerea* in different conditions *in vitro*. The ability to inhibit the growth of the fungus was tested in co-cultures, and with the interaction of only volatile or diffusible molecules produced by the bacterial strains. Both strains 260-02 and DC3000 managed to reduce the growth of BC in dual-culture assays, although with varying efficacy. Strain 260-02 gave 70% GIP, while DC3000 determined 56% GIP, results that differ significantly (Figure 2A).

In the dual-plate assay, strain 260-02 exhibited a higher inhibition rate than the one obtained with the aforementioned dual-culture assay (GIPv = 85.37%). This result could imply that diffusible components produced by bacteria could hinder the antifungal activity of the strain’s bouquet of VOCs, or elicit a defense response from the fungi.

Strain DC3000 showed no ability to inhibit BC through VOC production (GIPv = 0%) (Figure 2B).

In contrast with the aforementioned results, the solid media plates containing 50% cultural filtrate from strain DC3000 severely hampered BC growth, with a GIPf of 97%. The cultural filtrate of strain 260-02 had a less incisive, but still effective inhibition, with a GIPf of 91% (Figure 2C).

While this result confirms that both strains produce antifungal diffusible molecules, it cannot be compared to the dual-culture result, as the different values could be the result of different molecule concentrations, or of earlier direct contact between molecules and fungus, instead of the time lapse due to diffusion from the point of bacterial inoculum to the fungus. These results suggested that the main difference in the antifungal activity of strains 260-02 and DC3000 lies in their volatile molecule production, which was then investigated by SPME-GC-MS.

This experiment detected a wide array of molecules, see Table 2. Two interesting, and unexpected, observations can be made: (i) there is no significant difference for any compound between the vials containing only strain 260-02 or those with strain 260-02 plus BC, suggesting that the production of volatiles from the fungal strain is negligible, and that fungal presence does not influence strain 260-02’s volatiles production; and (ii) the differences in the volatile emission profile of strains 260-02 and DC3000 are few and, in most cases, strain DC3000 has a higher or at least an equivalent production of the compound, producing 4 more compounds than strain 260-02. This result highlights a complex molecular scenario that was not anticipated, and makes it hard to hypothesize a clear mode of action based on fungal inhibition through volatile molecules. The only compound present in a significantly higher amount in strain 260-02 is 1-4-octadiene, the data suggesting this molecule to be important for the antifungal effect, although it is not currently reported as an antifungal VOC (Piechulla et al., 2017). Furthermore, its effect as a discrete compound needs further investigation before drawing such a conclusion. Another possible interpretation is that one or more of the 4 compounds produced by strain DC3000 can either inhibit the effects of the active antifungal compounds or could be recognized by the fungus, causing it to stimulate its defense mechanisms. Another consideration is that the evaluation of the VOCs was carried out at an end-point situation, precluding any indication of the dynamics involved in the release of these molecules. Indeed, the order of the emission of these molecules, as well as the timing and speed with which they are emitted, could change the outcome of this volatile interaction tremendously, even though the final results are not so drastically different (Piechulla et al., 2017). Despite the fact that the VOC profiles were almost identical, the differences in the two strains are relevant enough to be visualized through a principal component analysis (PCA) carried out on the abundance of each compound (Figure 2D). Indeed, the volatiles discriminating between the two *P. syringae* strains on the second principal component are the four present only in strain DC3000.

**TABLE 2:** List of volatile molecules produced by the TGYA medium without inoculation, BC, strain DC3000, strain 260-02 and strain 260-02 in presence of BC, as detected through SPME-GC-MS.

**Molecule name**	**Quant**	**RT**	**TGYA**	**BC**	**DC3000**	**260-02**	**260-02+BC**
3-methyl butanal	41	7.17	13.41	12.5	–	–	–
1-undecene	41	16.44	–	–	15.28	15.56	15.5
Acetone	43	5.35	$12.21{}^{a}$	$13.74{}^{c}$	$12.95{}^{\mathrm{ab}}$	$13.06{}^{\mathrm{ab}}$	$13.35{}^{b}$
2-butanone	43	6.87	$12.66{}^{\mathrm{ab}}$	$11.75{}^{a}$	$12.25{}^{\mathrm{ab}}$	$13.23{}^{b}$	$12.80{}^{b}$
2-pentanone	43	9.45	$11.70{}^{a}$	–	$12.82{}^{b}$	$13.75{}^{c}$	$13.27{}^{\mathrm{bc}}$
Methyl isobutyl ketone	43	10.82	–	–	$11.73{}^{a}$	$12.64{}^{b}$	$12.07{}^{\mathrm{ab}}$
2-heptanone	43	18.73	–	–	$12.83{}^{a}$	$12.38{}^{a}$	$11.89{}^{b}$
2-nonanone	43	25.89	–	–	$14.40{}^{a}$	$12.51{}^{b}$	$12.42{}^{b}$
2-nonanol acetate	43	27.89	–	–	11.13	–	–
2-undecanone	43	31.57	–	–	$12.93{}^{a}$	$10.53{}^{b}$	$10.44{}^{b}$
3-OH-2-butanone	45	22.82	–	–	13.09	–	–
2-nonanol	45	29.54	–	–	13.30⁢a	11.70⁢b	11.65⁢b
Methyl undecyl ether	45	33.93	–	–	12.67⁢a	–	–
3-Methyl-1-butanol	55	20.14	$2.88{}^{a}$	$13.61{}^{b}$	$11.98{}^{b}$	$13.35{}^{b}$	$13.36{}^{b}$
1-Butanol	56	17.88	–	–	11.57	–	–
2-Methyl-1-butanol	57	20.08	$2.54{}^{a}$	$12.26{}^{b}$	$11.36{}^{b}$	$12.06{}^{b}$	$12.01{}^{b}$
1-4-octadiene	67	18.19	–	–	$11.63{}^{a}$	$12.11{}^{b}$	$11.78{}^{\mathrm{ab}}$
Cyclodecene	67	25.11	–	–	$10.50{}^{a}$	$9.81{}^{b}$	$9.51{}^{b}$
Phenylethyl alcohol	91	36.87	$9.50{}^{a}$	$12.40{}^{b}$	$12.29{}^{b}$	$12.57{}^{b}$	$12.38{}^{b}$
Dimethyl disulfide	94	13.9	$12.04{}^{\mathrm{ab}}$	$11.59{}^{a}$	$12.59{}^{\mathrm{ab}}$	$13.43{}^{\mathrm{ab}}$	$13.63{}^{b}$
Benzaldehyde	105	29.77	$15.25{}^{a}$	$10.02{}^{d}$	$9.84{}^{\mathrm{cd}}$	$8.80{}^{\mathrm{bc}}$	$8.45{}^{b}$
2-5-dimethyl pyrazine	108	24.03	$11.83{}^{\mathrm{bc}}$	$10.88{}^{a}$	$12.48{}^{c}$	$11.14{}^{\mathrm{ab}}$	$10.75{}^{a}$
3-ethyl 2,5 dimethyl pyrazine	135	27.7	$10.07{}^{\mathrm{ab}}$	$7.05{}^{a}$	$12.32{}^{b}$	$9.47{}^{\mathrm{ab}}$	$9.27{}^{\mathrm{ab}}$

*In the first column, a name of the molecule is reported, in the second column the quant ion specific for each compound is reported, in the third column the retention time of the molecule is reported. In the fourth, fifth, sixth, seventh, and eighth columns, the mean value of the natural logarithm of the peak area of each VOC is reported for TGYA, BC, DC3000, 260-02, and 260-02+BC treatments, respectively. Each value reported is the average obtained from 3 replicates of the experiment. Significantly different values according to One Way ANOVA, followed by Tukey’s Exact *post hoc* (*p* < 0.05), in each row are identified with different letters.*

### Biocontrol Against *B. cinere*a

**FIGURE 3:**
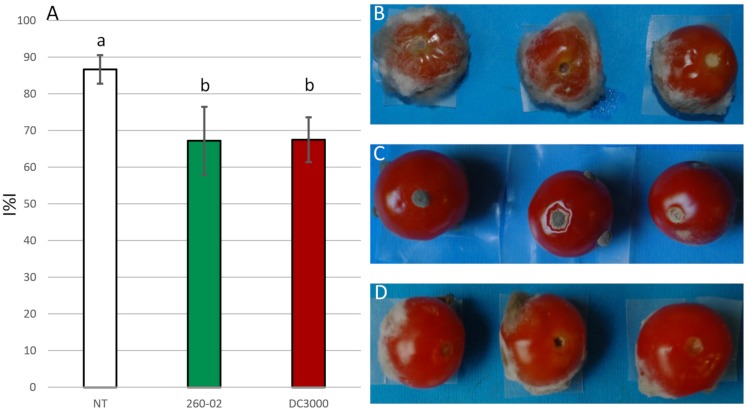
Results of the *in vivo* assays. **(A)** Graph reporting the percentage infection index (I%I) observed on tomatoes inoculated with BC alone (NT), or previously treated with either strain 260-02 or strain DC3000. The bars indicate the mean I%I value observed on three replicates of the experiment, each carried out on 10 fruits. Different letters (a,b) indicate significantly different results according to One-Way ANOVA test, followed by Tukey’s Exact *post hoc* (*p* < 0.05). On the right side, pictures of three representative tomatoes that were **(B)** non-treated, **(C)** treated with strain 260-02, and **(D)** treated with strain DC3000.

The ability of the bacterial strains 260-02 and DC3000 to inhibit BC growth was also assayed on tomatoes, punctured and inoculated with BC spores and cell suspensions of each strain. Both strains, 260-02 and DC3000, resulted positive, significantly reducing the I%I, compared to the non-treated control (I%I = 92), to values of 67 and 75, respectively (Figure 3A), as seen in the pictures of the treated and non-treated tomatoes (Figures 3B–D). No significant difference could be observed in the two strains in this assay. This result is in line with earlier results: the only known example of *P. syringae* strains used for biocontrol is to antagonize *B. cinerea*. While there are few details regarding the use of *P. syringae* as a biocontrol agent, it is known that a registered biocontrol product utilizes one or more *P. syringae* strains capable of competing for colonization with *B. cinerea*, therefore reducing the symptoms caused by this pathogen (Haidar et al., 2016).

### Plant-Growth Promotion

**FIGURE 4:**
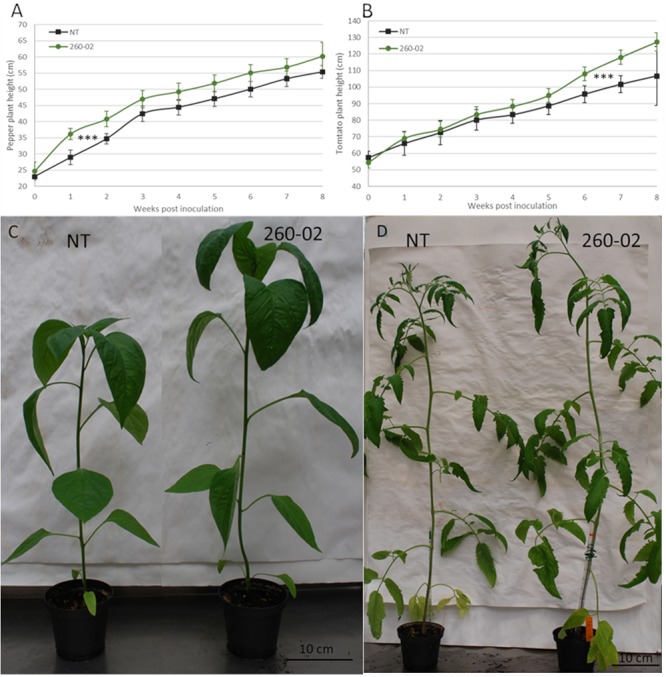
Results of the *in planta* PGP assay. Graphs reporting the average height of the plants, 7 replicates per treatment, either non-treated (NT), or treated by root dipping with strain 260-02, for panel **(A)** pepper plants and for panel **(B)** tomato plants. The vertical axis reports the height in centimeters of the plants, while the horizontal axis reports the weeks from the treatment with the strain. The asterisks (^∗∗∗^) indicate that for both experiments, the progression of height throughout the 8 weeks found to be statistically different (*p* < 0.001) according to a general linearized model test, optimized for repeated measures. On the lower side, pictures showing the non-treated and 260-02-treated plants of panel **(C)** pepper and panel **(D)** tomato one month after treatment.

The effect of strain 260-02 on pepper and tomato plant growth was assayed in a greenhouse experiment, the plants, at transplant, being root-dipped in a suspension of strain 260-02, and their height monitored. Pepper plants inoculated by root-dip with strain 260-02:*gfp* showed a quick response in terms of height, becoming significantly taller than the non-treated plants as early as 1 week after inoculation (Figure 4A). This increase in height remained consistent until the late stages of growth, the non-treated plants catching up with the growth of the 260-02:*gfp* treated plants, though there was still a statistically significant difference in height. The increase in height of the treated peppers was around 20%, in line with earlier results obtained with growth promoters (Kang et al., 2007; Zhang et al., 2016).

For the tomato plants, the opposite happened: the treated and non-treated plants showed very similar growth patterns until the last 3 weeks of monitoring, when the 260-02:*gfp* inoculated plants showed greater height development than the non-treated (Figure 4B). Both 260-02:*gfp* inoculated pepper and tomato plants flowered earlier, the flowers developing approximately 7–10 days ahead of the non-treated controls, a phenomenon already reported for other well-known plant-growth promoter bacteria such as *Paraburkholderia phytofirmans* strain PsJN (Wang et al., 2015). A visual comparison of the NT and 260-02-inoculated plants is shown in Figure 4C for pepper plants and Figure 4D for tomato.

### Biocontrol Against *P. syringae* pv. *tomato* DC3000

**FIGURE 5:**
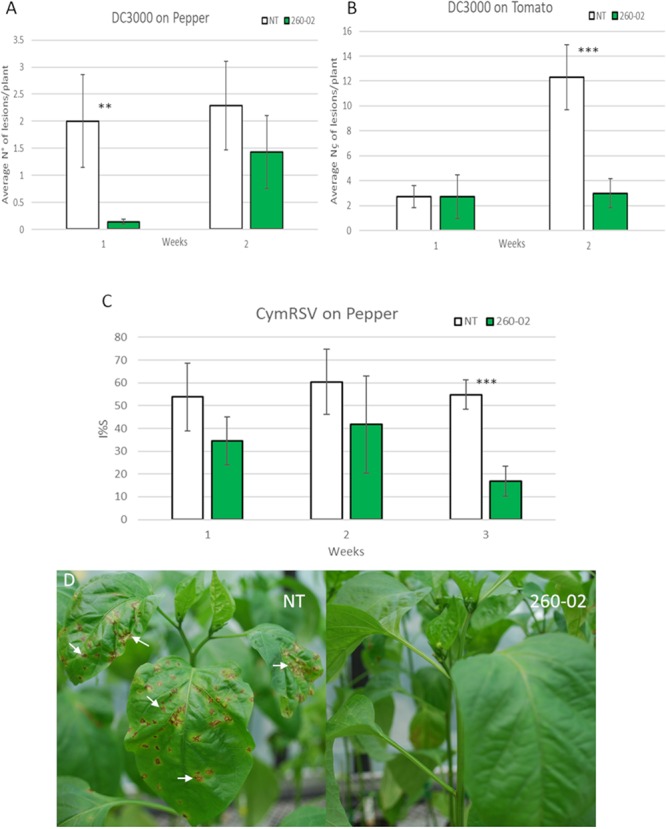
Results of the *in planta* biocontrol assays. Green bars indicate the plants that were treated with strain 260-02 by root dipping 2 weeks (for biocontrol against DC3000) or one month (for biocontrol against *Cymbidium Ringspot Virus*) before being challenged with the pathogen, while white bars indicate non-treated control plants. For each combination of treatment, pathogen, and host, 7 biological replicates were used. **(A)** graph reporting the number of lesions developed by *P. syringae* strain DC3000 on pepper plants; **(B)** graph reporting the number of lesions developed by *P. syringae* strain DC3000 on tomato plants. The vertical axis shows the average number of lesions per plant, on the horizontal axis are divided the observations 1 and 2 weeks after inoculation with the pathogen. Statistically different results according to the Student’s *T*-test are reported with asterisks (^*^p < 0.05; ^∗∗^*p* < 0.01; ^∗∗∗^*p* ≤ 0.001). **(C)** graph reporting the percentage infection index (I%I) registered on pepper plants inoculated with *Cymbidium Ringspot Virus* (CymRSV). Statistically different results according to Student’s *T*-test are reported with asterisks (^*^p < 0.05; ^∗∗^*p* < 0.01; ^∗∗∗^*p* ≤ 0.001). **(D)** Pictures of representative plants that were inoculated with CymRSV: typical symptoms of the disease can be seen on the leaves of the non-treated plant, with evident circular necrotic spots; the plants also inoculated with strain 260-02 show fewer or, as reported in the picture, no symptoms on newer leaves.

The biocontrol ability of strain 260-02 to act against a phytopathogenic *P. syringae* strain was tested. Tomato and pepper plants, either mock-inoculated or inoculated at the root with *P. syringae* pv. *syringae* strain 260-02, were inoculated on the leaves with *P. syringae* pv. *tomato* strain DC3000. Both plant species that had root treatment with strain 260-02:*gfp* showed less susceptibility to the pathogen. All the pepper plants, with and without treatment, showed relatively few lesions, but 1 week after pathogen inoculation, the 260-02:*gfp*-treated plants showed even fewer, and less severe lesions, having developed on average, less than one lesion per plant. After another week the number of lesions was even fewer, although not significantly so (Figure 5A). Tomato plants, both treated and non-treated, showed the same level of infection 1 week after inoculation, while after 2 weeks the non-treated plants developed more severe symptoms, the infection on the 260-02:*gfp*-inoculated plants remaining stable with very few new lesions being developed (Figure 5B).

### Biocontrol Against *Cymbidium Ring Spot Viru*s

The ability of strain 260-02 to provide a biocontrol effect against a phytopathogenic virus was tested. Pepper plants, either mock-inoculated or inoculated at root level with strain 260-02, were mechanically inoculated on the leaves with CymRSV. The inoculated pepper plants all showed severe symptoms on the directly inoculated leaves, the leaves developing circular necrotic spots, yellowing, and ultimately leaf detachment. Instead the symptoms of systemic virus infection were very different for the 260-02:*gfp*-treated plants and the non-treated controls, as the percentages reported in Figure 5C. While all three measurements showed the infection to be consistently lower, the last one showed a significant difference between the treated and control plants. This was mostly determined by two 260-02:*gfp*-treated plants that showed no symptomatic leaves, the plants appearing healthy (Figure 5D). Since there is no known direct biocontrol method that reduces viral pathogen symptoms, the reduced CymRSV symptoms found in the 260-02:*gfp*-treated plants suggest that the strain induces host resistance to pathogens. It would be interesting to investigate this phenomenon further, in order to elucidate which of the host’s pathways are affected and which molecules of the strain are responsible for this effect.

### Colonization Assays

Pepper and tomato seedlings were inoculated, either at the root and/or on the leaves, with *gfp*-labeled mutants of either strain 260-02 or DC3000. Then the colonization patterns of roots and leaves were compared for these two *P. syringae* strains, by visualizing the GFP fluorescence on the surface of the treated plants.

Representative pictures of each treatment, on both pepper and tomato plants, at 3 and 6 dpi are provided to show the phenotype of the inoculated plants (Supplementary Figure S1). Small lesions can be seen forming at 6 dpi on plant leaves inoculated with strain DC3000, while no symptoms are visible on those inoculated with strain 260-02.

Visualization through confocal microscopy showed how the two strains colonize the same host on the plant’s surface. For brevity and clarity, the presented images are from pepper plants at 3 dpi, while all the other images regarding tomato plants and the different time points, are available in Supplementary File 1.

The negative control plants showed no GFP fluorescence, confirming that the GFP signal detected in the treated plants corresponds to the transformed strains inoculated in the host (Supplementary File 1).

**FIGURE 6:**
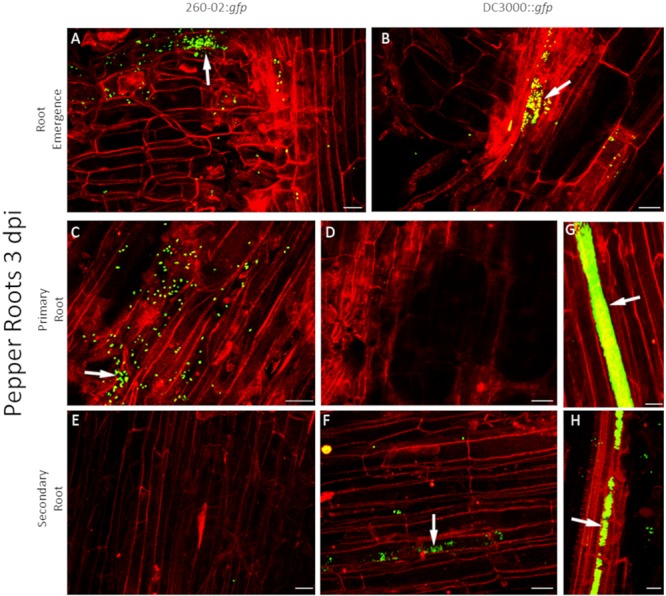
Confocal microscopy on pepper roots, 3 days after inoculation with bacterial strains. The panel shows representative pictures from the microscopy observations. In green is the fluorescence obtained by exciting with a wavelength of 488 nm, which produces fluorescence from the GFP used to tag the bacterial strains, in red that obtained by exciting with a wavelength of 594 nm, which produces autofluorescence from phenolic compounds and allows to visualize plant material, in particular cell walls. Yellow color is given by overlap of red and green fluorescence. White arrows indicate examples of GFP fluorescence on the root surface. Panels **(A,C,E)** are taken from plants inoculated with strain 260-02:*gfp*; panels **(B,D,F–H)** are taken from plants inoculated with strain DC3000::*gfp*. Panels **(A,B)** portray zones of emergence of secondary roots; panels **(C,D,G)** portray primary roots; panels **(E,F,H)** portray secondary roots. In each picture, the scale bar reported in the lower right corner corresponds to 20 μm.

For root colonization, the two strains show some similarities and some differences. Both strains were capable of colonizing both assayed host species, and showed a preference for colonization at the emergence of a secondary root (Figures 6A,B). Strain 260-02:*gfp*, but not strain DC3000, was found often, and in high numbers, on the surface of primary roots (Figures 6C,D), whereas strain DC3000::*gfp* was found more often on secondary roots, where strain 260-02:*gfp* was hardly present (Figures 6E,F). Strain DC3000::*gfp* showed extensive colonization of damaged roots, and tended to locate over the xylem zones, evidenced by solid green bands in the middle of the roots (Figures 6G,H). Strain 260-02:*gfp* did not show such behavior.

Neither strain showed a preference for root tip colonization, this occurring only with very scarce bacteria presence, if at all (Supplementary File 1E). This similar, but distinct behavior of the two strains could relate to different interactions with the plant: it is known that both plant pathogens and plant beneficial bacteria must overcome plant defenses to successfully establish themselves in the host. At the root level, an important step of this interaction is a weakening of plant defenses by negative regulation of salicylic acid-mediated response pathways (Millet et al., 2010). With regard to the pathogenic strain DC3000, it is known that this weakening is related to the production of coronatine when interacting with plant roots (Millet et al., 2010), whereas for plant-beneficial bacteria, such as fluorescent *Pseudomonas*, the molecule responsible is currently unknown. Considering the different colonization pattern of the two examined strains, it is possible to hypothesize a similar difference in molecular cross-talk between bacteria and plants.

The assay to compare leaf colonization allowed the comparison of the two strains during interaction with the two host species, and also an evaluation of the effect of root colonization on the establishment of the pathogenic strain DC3000::*gfp*.

A visual investigation of the leaves showed no macroscopic symptoms developing on untreated leaves or leaves treated with strain 260-02, and few, or no symptoms on leaves treated with the pathogenic strains DC3000 and DC3000::*gfp* (Supplementary Figure S1). This could be due to the short time between inoculation and symptom assessment, still it does confirm that transformation with a fluorescent protein did not inhibit the strain’s ability to act as a pathogen, and that strain 260-02 is non-pathogenic on tomato and pepper in the experimental conditions used.

**FIGURE 7:**
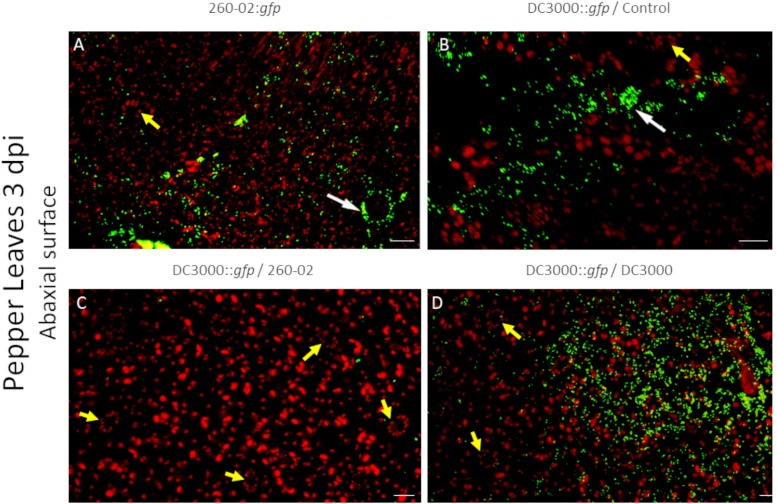
Confocal microscopy on the abaxial surface of pepper leaves at 3 dpi. The panel shows representative pictures from the microscopy observations. In green is the fluorescence obtained by exciting with a wavelength of 488 nm, which produces fluorescence from the GFP used to tag the bacterial strains, in red that obtained by exciting with a wavelength of 594 nm, which produces autofluorescence from chlorophyll and visualizes the position of chloroplasts. Yellow color is given by overlap of red and green fluorescence. Panel **(A)** is from leaves experimentally inoculated with a suspension of strain 260-02:*gfp* cells; panel **(B)** is from leaves experimentally inoculated with a suspension of strain DC3000::*gfp* cells taken from plants without any root treatment; panel **(C)** is from leaves experimentally inoculated with a suspension of strain DC3000::*gfp* cells taken from plants treated at the root with strain 260-02; panel **(D)** is from leaves experimentally inoculated with a suspension of strain DC3000::*gfp* cells taken from plants treated at the root with strain DC3000. White arrows point to stomata being colonized by bacterial cells, visible as green outlines of the guard cells and the stoma aperture; yellow arrows point to non-colonized stomata, visible as oval patterns in chloroplast position with a dark area in the middle. In each picture, the scale bar reported in the lower right corner represents 20 μm.

**FIGURE 8:**
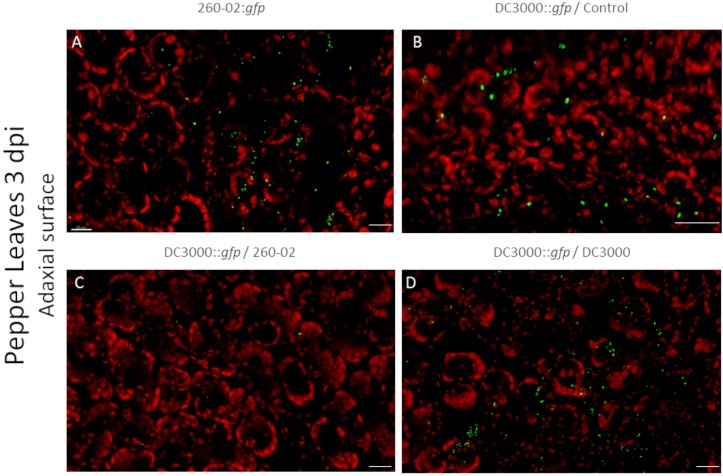
Confocal microscopy on the adaxial surface of pepper leaves at 3 dpi. The panel shows representative pictures from the microscopy observations. In green is the fluorescence obtained by exciting with a wavelength of 488 nm, which produces fluorescence from the GFP used to tag the bacterial strains, in red that obtained by exciting with a wavelength of 594 nm, which produces autofluorescence from chlorophyll and visualizes the position of chloroplasts. Yellow color is given by overlap of red and green fluorescence. Panel **(A)** is from leaves experimentally inoculated with a suspension of strain 260-02:*gfp* cells; panel **(B)** is from leaves experimentally inoculated with a suspension of strain DC3000::*gfp* cells taken from plants without any root treatment; panel **(C)** is from leaves experimentally inoculated with a suspension of strain DC3000::*gfp* cells taken from plants treated at the root with strain 260-02; panel **(D)** is from leaves experimentally inoculated with a suspension of strain DC3000::*gfp* cells taken from plants treated at the root with strain DC3000. In each picture, the scale bar reported in the lower right corner represents 20 μm.

Also in this case, the two strains had similarities and differences, and were able to colonize both host plants. Both strains showed a preference for colonization of the abaxial surface, particularly the stomata, as expected (Figures 7A,B). The adaxial surface of the leaves showed only sporadic colonization, with few fluorescent colonies visible (Figures 8B,C). The difference was extremely noticeable in plants treated with strain 260-02:*gfp*, and with those treated with strain DC3000::*gfp* on the leaves after root treatment with strain DC3000 (Figures 8A,D), compared to the abaxial surface of the same leaves (Figures 7A,D). Also, the bacterial cells were visualized at the level of the epidermis cells on the abaxial surface of the leaf, while they localized in the mesophyll of the adaxial surface, suggesting different colonization preferences in the different surfaces of the leaf.

From a visual observation of the leaves, it seems that strain DC3000::*gfp* colonization is less widespread than that of strain 260-02, the former establishing itself only in limited portions of the leaves, and often with lesion development, while the latter is established on wider portions of the leaves, and without lesions. The early symptoms caused by strain DC3000 could not always be seen by visually examining the leaves at 3 dpi, but they were visible under microscope magnification: circular areas characterized by the lack of red autofluorescence in epifluorescence, that instead showed an intense red fluorescence when excited at 594 nm and colonization by strain DC3000::*gfp*.

**FIGURE 9:**
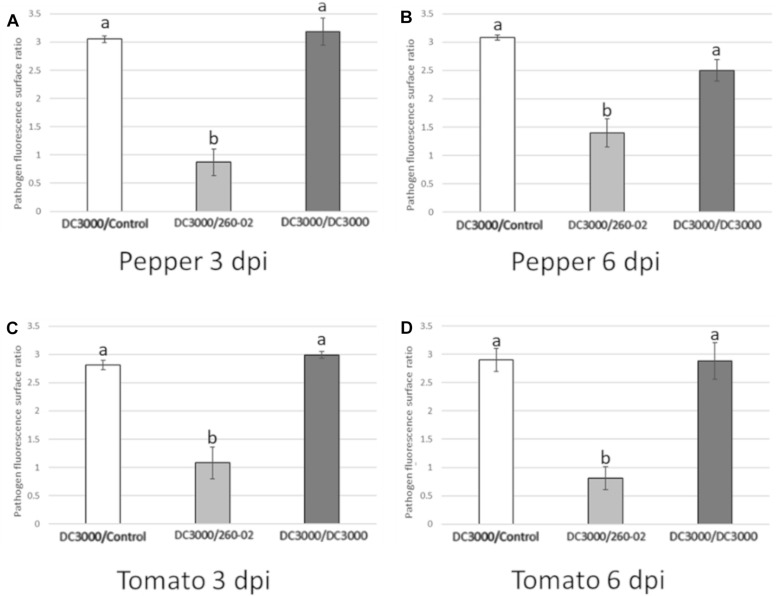
Charts reporting the results of the quantitative analyses of confocal microscopy pictures taken from the abaxial surface of leaves infected by DC3000::*gfp* (Figure 7) either with PBS root treatment (DC3000/Control, in white), with 260-02 root treatment (DC3000/260-02, in light gray), or with DC3000 root treatment (DC3000/DC3000, in dark gray). On the vertical axis is the amount of leaf surface, expressed as a base 10 logarithm of μm^2^, on which GFP fluorescence from the bacteria was visible. Error bars indicate standard deviation. Bars represent the average value obtained by analyzing 7 images per treatment. Letters (a,b) indicate significantly different results according to One-Way ANOVA test, followed by Tukey’s Exact *post hoc* (*p* < 0.05). Graphs **(A,B)** indicate results obtained in pepper plants at 3 and 6 dpi, respectively. Graphs **(C,D)** indicate results obtained in tomato plants at 3 and 6 dpi, respectively.

Quantitative analyses carried out using confocal microscopy pictures allowed the quantification of the different root treatments on the colonization ability of strain DC3000::*gfp* on the host leaves. Plants that were mock-treated or treated with strain DC3000 showed comparable results in both hosts and at both time points, with an average of approximately 1000 μm^2^ of each examined leaf showing GFP fluorescence, representing around 1.5% of the examined surface, while plants that were treated with strain 260-02 showed an average of approximately 10 μm^2^, representing less than 0.1% of the examined surface (Figure 9). This difference is statistically significant and, having been determined by a distal inoculation, suggests that strain 260-02 is capable of inducing host resistance against pathogens, and does not reduce the symptoms by direct interaction with the pathogens. These results are in accordance with the reduction of symptom severity observed in the biocontrol assay against strain DC3000, after inoculation at the roots with strain 260-02 (Figures 5A,B).

### Comparative Pangenomic Analysis

The pangenome analysis carried out in this study compared the gene content of nine genomes from pathogenic *P. syringae* strains, one genome from pathogenic *P. savastanoi* strain, and the genome of strain 260-02, with the aim of characterizing which genes differentiate the pathogenic strains from the beneficial one.

The output of the pangenome analysis was manually analyzed to detect the genes that were present in pathogenic strains but not in strain 260-02, and the genes that differentiated strain 260-02 from strains UMAF0158, its pathogenic closest relative, and strain DC3000, which was used as a model organism to compare behavior on Solanaceae crops in experimental trials. Furthermore, the genes that were reported as being present exclusively in 260-02 and not in the pathogenic strains were searched for.

Without any information apart from their presence or absence, genes identified as “hypothetical protein” were not considered in the present study, though along with other genes their numbers are reported in Table 3. These genes, which represent most of the non-shared genes, with over 5,000 of them not present in strain 260-02, could possibly be very important for pathogenicity but, at the moment, they cannot be analyzed through bioinformatics due to the lack of a proper functional characterization.

**TABLE 3:** The number of genes that are present in analyzed pathogenic strains, and not in strain 260-02.

**Gene category**	**Absent in 260-02 compared to pangenome strains**	**Absent in 260-02 compared to *P. syringae* UMAF0158**	**Absent in 260-02 compared to *P. syringae* DC3000**	**Absent in 260-02 and common in UMAF0158 and DC3000**
Toxin/Effectors	40	2	16	1
Quorum sensing	18	2	7	0
SS Type II	15	0	0	0
SS Type III	14	0	8	0
SS Type IV	17	7	2	1
SS Type VI	1	0	1	0
Gene expression	8	1	2	0
Hypothetical	5,531	285	1,160	0
Other	4	0	2	0

*The first column indicates the category these genes fall into, if they have a known function, or Hypothetical if they do not. The second column indicates the total number of genes absent in strain 260-02 when compared to all the other genomes used in the analysis. The third and fourth column indicate the number of genes absent in strain 260-02 when compared to either its closest relative, strain UMAF0158, or the model pathogen strain DC3000. The last column indicates the number of genes that are absent in strain 260-02 that are instead present in both UMAF0158 and DC3000 strains.*

#### Genes Absent From the Genome of *P. syringae* pv. *syringae* 260-02

In total, 117 genes with function prediction are absent in strain 260-02 (Supplementary Table S2), and the genes are grouped into seven different functions (Table 3), the highest number of genes (40) belonging to the functional category of toxins and effectors. Two of these genes were identified in strain UMAF0158, and could therefore be important determinants for pathogenic behavior: (i) ornithine aminotransferase, which could be related to the production of typical *P. syringae* toxins that interfere with the synthesis of arginine by inhibiting an enzyme that works on the same substrate (ornithine N-acyl transferase) (Carrion et al., 2012) and (ii) virulence regulon transcription activator VirF, which gets its name from the system identified in animal pathogens, such as *Shigella flexneri*, is crucial to host invasion (Dorman et al., 2001), but could conceivably be related to the expression of virulence also in *P. syringae*. Interestingly, this gene is also present in the genome of strains DC3000 and UMAF0158, and could therefore be important for the determination of pathogenesis in *P. syringae*.

Additionally, strain 260-02 lacks 18 genes encoding for quorum sensing components (Table 2). Two of them were found in the genome of closest relative UMAF0158 and were identified as a gene for a specific *luxR* receptor and a homoserine/homoserine lactone efflux pump gene. *luxR* genes are traditionally associated with sensing acyl-homoserine lactones (AHL), mediating the response to quorum sensing, however, there are several genes of the *luxR* family that bind other molecules (Patel et al., 2013). Thus, it is difficult to draw conclusions on the function of this specific LuxR receptor and its role in determining the phenotype of plant–microbe interaction in *P. syringae*. Strain DC3000 possesses seven quorum-sensing genes that are not present in strain 260-02, all of which are related to the autoinducer system of quorum sensing. Of particular interest could be the *psyI* gene, homolog of *luxI* and responsible for the synthesis of AHL (Antunes and Ferreira, 2009), which hints at the possibility that strain 260-02 can perceive quorum sensing molecules but does not directly contribute to achieving the quorum threshold. Still, this gene is missing in strain UMAF0158, in an otherwise extremely syntenic region of the genome between the three organisms, which suggests that it is not strictly necessary for the development of pathogenicity.

In total, 47 genes encoding for components of different secretion systems (Type II, Type III, Type IV and Type VI) in the pangenome of *P. syringae* were not found in the genome of strain 260-02 (Table 3). Interestingly, the 14 Type III secretion system related genes absent from the genome of strain 260-02 are also missing in strain UMAF0158. Consequently, although the Type III secretion system is well-known as a virulence factor in *P. syringae* (Mudgett, 2005; Tang et al., 2006; Lindeberg et al., 2012) none of these genes might be strictly necessary for the pathogenicity of *P. syringae* strains. Of these 14 genes, eight are present in strain DC3000, mostly effector proteins secreted through the type III secretion system. This is in line with the extremely redundant nature of Type III effectors, as recent studies conducted on this class of molecules demonstrated that no single effector is strictly necessary for pathogenicity, or determines host range, therefore very few of these factors are strictly conserved (Guttman et al., 2014). Therefore, it is possible that the lack of these specific Type III effectors contributes to the non-pathogenic behavior, but are not strict determinants.

In contrast, seven genes encoding for the Type IV secretion system components absent in strain 260-02 are present in strain UMAF0158, indicating that these genes could have a direct impact on pathogenicity. These genes mostly encode for different VirB proteins, which are important determinants for the virulence in *Agrobacterium tumefaciens* (Berger and Christie, 1994), but such a role is unknown for *P. syringae*. One of these genes is also present in the genome of strain DC3000 and encodes for a precursor of the VirB5 protein, supporting the hypothesis that it could be important for pathogenesis.

One Type VI secretion system gene encoding for a lipoprotein was absent from the genome of strain 260-02 but also from strain UMAF0158, hinting that this gene might not be involved in the determination of the phenotype of plant–microbe interaction in *P. syringae*.

Furthermore, strain 260-02 lacks eight genes representing transcription activators and methylation proteins (Table 3). Of these genes, two are present in the genome of strain DC3000, one is identified as a transcriptional repressor but does not have a specific pathway associated with it, the other is identified as a *virS* gene, which is known to be part of a two-component regulator system of toxin production in *Clostridium perfringens* (Ba-Thein et al., 1996). The relevance of this system is not known for pathogenicity of *P. syringae*, but this system could be involved in pathogenic behavior, though not strictly necessary. Only one of these eight genes is present in UMAF0158, but not in 260-02, a DpnIIA modification methylase. Methylation of adenine is known to be part of the resistance mechanisms against exogenous DNA in bacteria: bacteria employ several restriction enzymes to degrade exogenous DNA, and also produce methylases that will protect non-target DNA from degradation. Therefore, adenine methylation ultimately regulates the acquisition of exogenous DNA in bacterial cells (Johnston et al., 2013). Furthermore, adenine methylation has been shown to play a key role in the regulation of pathogenesis in animal pathogens such as *Klebsiella pneumoniae* (Fang et al., 2017). Therefore, having different genes related to the methylation of adenine could cause a difference in pathogenic behavior.

Four genes clustered in the category “others” and encoding an acetoin catabolism regulatory protein, a benzaldehyde dehydrogenase, and two different pesticin receptor precursors. All of these genes are present in DC3000 while none is present in UMAF0158, and they are therefore unlikely to be important determinants of pathogenicity.

#### Genes Exclusively Found in the Genome of *P. syringae* pv. *syringae* Strain 260-02

The pangenomic analysis highlighted the presence of 318 found exclusively in the genome of strain 260-02, but absent in all other *P. syringae* genomes analyzed in this study. Of these genes, only 8 had a function assigned to them, while the remaining 310 encoded for either hypothetical proteins, or proteins of unknown function.

Of these eight genes, four encode for defense-related functions with three being involved in arsenate resistance and one in protection against the bacteriocin colicin. Of the remaining four genes, one encodes for a chitinase, which could be related to an antifungal activity of the bacterium, even if it was not highlighted during the *in vitro* antifungal assays.

The last three genes are the ones that seem the most interesting, being three DpnIIA modification methylases (gene IDs 1238, 1735, 4148) that are different from other proteins in this class that are present in other *P. syringae* genomes.

The DpnIIA-encoding gene 1238 has high similarity to a methylase present in *P. syringae* pv. *japonica* (90% cover, 98% identity on the AA sequence), but finds no other match in the *Pseudomonas* genus and, interestingly, a BLAST analysis of the nucleotide sequence gives only one match (cover 9%, identity 76%) with the genome of *Desulfotomaculum reducens* strain MI-1. Utilizing the discontiguous megablast algorithm instead of the regular megablast; there is a single match within the *Pseudomonas* genus on the genome of *P. psychrotolerans* strain PRS08-11306 (coverage 93%, identity 72%).

The DpnIIA-encoding gene 1735 shares only 80% similarity at the AA level with other proteins of this class, and is the least similar at the nucleotide level, without any homology with other *P. syringae* genomes by BLAST.

The DpnIIA-encoding gene 4148 gene shares 95% similarity at the AA level with a single *P. syringae* strain (identified as Leaf127), but has no other homologous sequence in the *Pseudomonas* genus, with the next most similar protein, sharing 79% identity, being identified in *Burkholderia* sp. strain CF099.

Furthermore, all three genes are flanked by proteins that are identified as being of viral origin, suggesting that they have been introduced in the genome by horizontal gene transfer by phages, rather than being part of the gene pool of wild *P. syringae*.

Taking into consideration the aforementioned role of adenine methylation in the development of pathogenesis in other pathogens, such as *K. pneumoniae* and *Staphylococcus pneumoniae* (Johnston et al., 2013; Fang et al., 2017), it is plausible that the presence of adenine methylase genes that have either different pattern recognition, or are not functional, could lead to a non-pathogenic phenotype.

## Conclusion

The present study investigates, in depth, the behavior of a beneficial *P. syringae* pv. *syringae* strain, in comparison with a model pathogen, *P. syringae* pv. *tomato* strain DC3000, at both a phenotypic and a genomic level.

Considering that *P. syringae* is well known worldwide as a pathogen, and is a model organism used to study host–pathogen interaction, the new information provided on this beneficial strain could be helpful for further studies aimed at understanding the differences between plant pathogens and beneficial microorganisms.

The differences between the two categories already appeared to be less than originally expected, due to recent studies, and the hypotheses that (i) bacteria are neither intrinsically pathogenic nor beneficial, but their plant-interaction potential could lead to either result based on either the host and/or the environmental conditions (Brader et al., 2017) and (ii) the different behavior of closely related strains is better explained by rather subtle differences, such as the expression of genes leading to plant defense reactions (Sheibani-Tezerji et al., 2015a), appear to be even more concrete in the light of these findings.

While strain 260-02 carries a mangotoxin operon (*Mbo*) that is closely related to a non-functional operon, possibly precluding the production of mangotoxins, it has many other known pathogenesis-related genes of *P. syringae*. For example, the genome of strain 260-02 encodes all the genes needed to produce a functional Type III Secretion System, and an array of effectors that could be injected into plant cells, as well as good colonization abilities on both roots and leaves. Considering these traits, it is difficult to consider strain 260-02 a “disarmed pathogen” (Reinhold-Hurek and Hurek, 2011), since, unlike many other plant-beneficial *Pseudomonas* strains previously described in literature, it still possesses the genes required to produce the most important weapons needed to act as a pathogen. By taking into account that the few genes with a known function unique to strain 260-02 are related to DNA methylation, our hypothesis is that the strain is, instead, a “pacifist pathogen”: it still possesses the weapons of a pathogenic strain, but the different methylation pattern does not allow it to use these weapons to harm the host.

From an evolutionary point of view, this behavior could lead to the main pathogenesis-related genes becoming pseudogenes and ultimately, in a long-term scenario, getting lost by the strain.

The greatest limit of the hypothesis, that the differences between plant-pathogenic and plant–beneficial interactions are mostly dictated by methylation patterns and differential gene expression in the strains, is the high number of genes of unknown function. While these genes could be completely unrelated to plant–microbe interactions, or even be non-coding, some could be involved in plant–microbe interactions (Sheibani-Tezerji et al., 2015b).

Since the precise genetic basis behind the non-pathogenic behavior of strain 260-02 was not determined as part of this study, this strain is not suitable for use as an actual biocontrol strain. Considering also the genome plasticity of the *P. syringae* species, strain 260-02 could switch to pathogenic behavior in the presence of other strains or through horizontal gene transfer, making its introduction into an ecosystem extremely dangerous. Despite this, strain 260-02 is an interesting organism to study, and opens new, interesting perspectives for future studies in the field of plant–microbe interactions.

## Author Contributions

AP, PC, and BM contributed concept and design of the study. AP, PC, SC, and GB contributed to design methods employed in the studies. MM, LA, and DS contributed with the use of software. AP and DS carried out statistical analyses. AP, ST, and GB performed acquisition of the data. PC, SC, FM, MD, MB, and BM provided resources used in the study. AP, SC, DS, and FQ contributed to the production of figures used in the manuscript. PC, MD, and BM supervised the study. AP wrote the first draft of the manuscript. All authors contributed to manuscript revision, and read and approved the submitted version.

## Conflict of Interest Statement

The authors declare that the research was conducted in the absence of any commercial or financial relationships that could be construed as a potential conflict of interest.
