# Supplementary material for: Not Just a Pathogen? Description of a Plant-Beneficial Pseudomonas syringae Strain
Source: Front Microbiol. 2019 Jun 21;10:1409. doi: 10.3389/fmicb.2019.01409 (PMC6598456; doi:10.3389/fmicb.2019.01409)
Supplement: Supplementary file 1 [file Image_1.pdf]

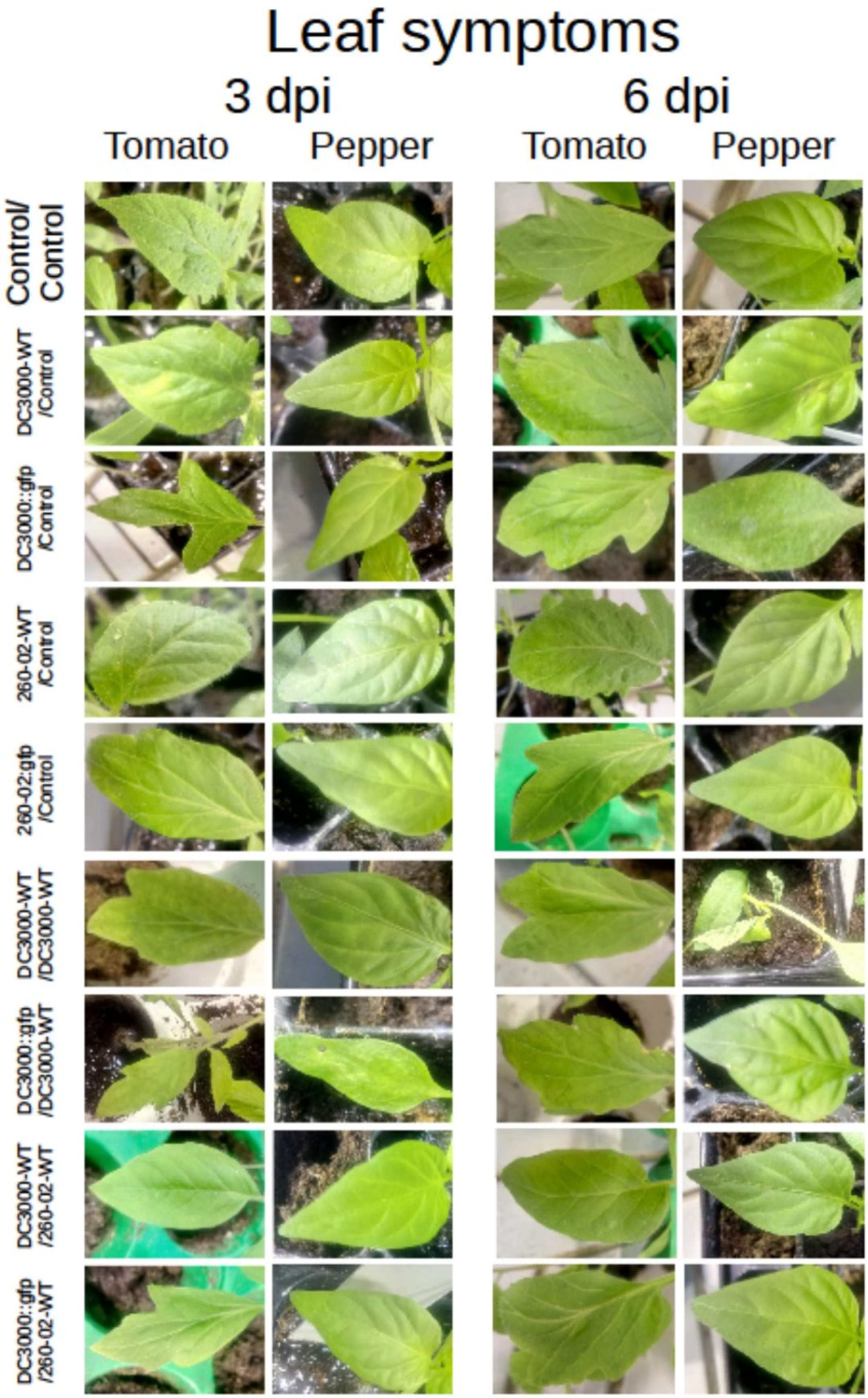

Supplementary Figure S1: pictures of leaves of pepper and tomato plants 3 and 6 days post inoculation. Each column represents one host at one time point, from left to right: tomato 3 dpi, pepper 3 dpi, tomato 6 dpi, pepper 6 dpi. Each row represents a different treatment, indicated on the Y-axis. The first line of the label indicates the leaf treatment (Control, 260-02 or DC3000, either wild type or mutants expressing GFP), while the second line of the label indicates the root treatment (Control, 260-02 or DC3000 wild-type).
