# Supplementary material for: Not Just a Pathogen? Description of a Plant-Beneficial Pseudomonas syringae Strain
Source: Front Microbiol. 2019 Jun 21;10:1409. doi: 10.3389/fmicb.2019.01409 (PMC6598456; doi:10.3389/fmicb.2019.01409)
Supplement: Supplementary file 2 [file Data_Sheet_1.PDF]

Control plant roots

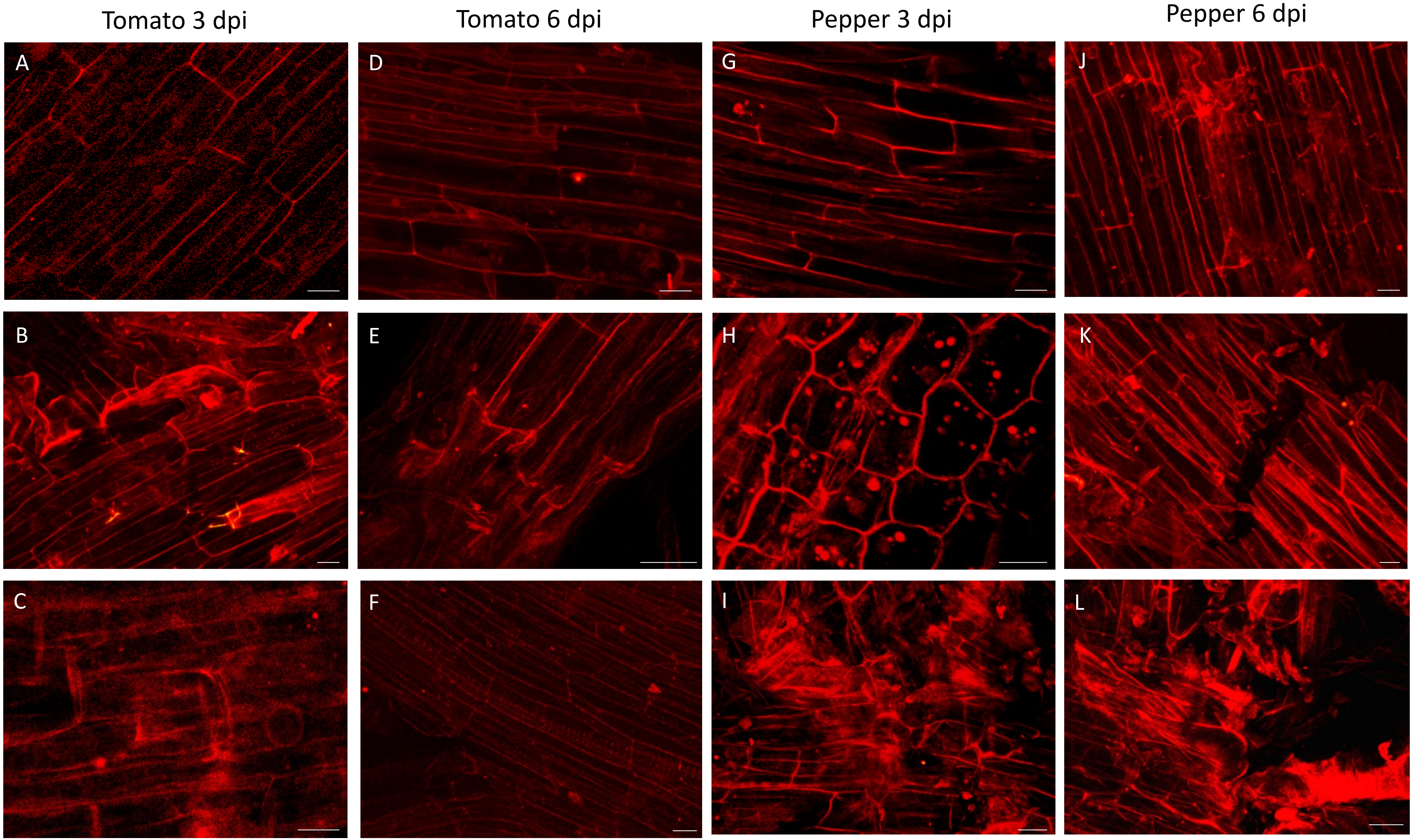

Supplementary file 1A
Confocal microscopy on non-treated tomato and pepper roots. The panel shows representative pictures from the microscopy observations on control plants. In green is the fluorescence obtained by exciting with a wavelength of 488 nm, which produces fluorescence from the GFP used to tag the bacterial strains, in red that obtained by exciting with a wavelength of 561 nm, which produces autofluorescence from plant tissues and visualizes plant material. Pictures from A to C were taken on tomato roots at 3 dpi, while those from D to F were taken on tomato roots at 6 dpi. Pictures from G to I were taken on pepper roots at 3 dpi, while those from J to L were taken on pepper roots at 6 dpi. In each picture, the scale bar reported in the lower right corner represents 20 µm.

Tomato plant roots 3 dpi

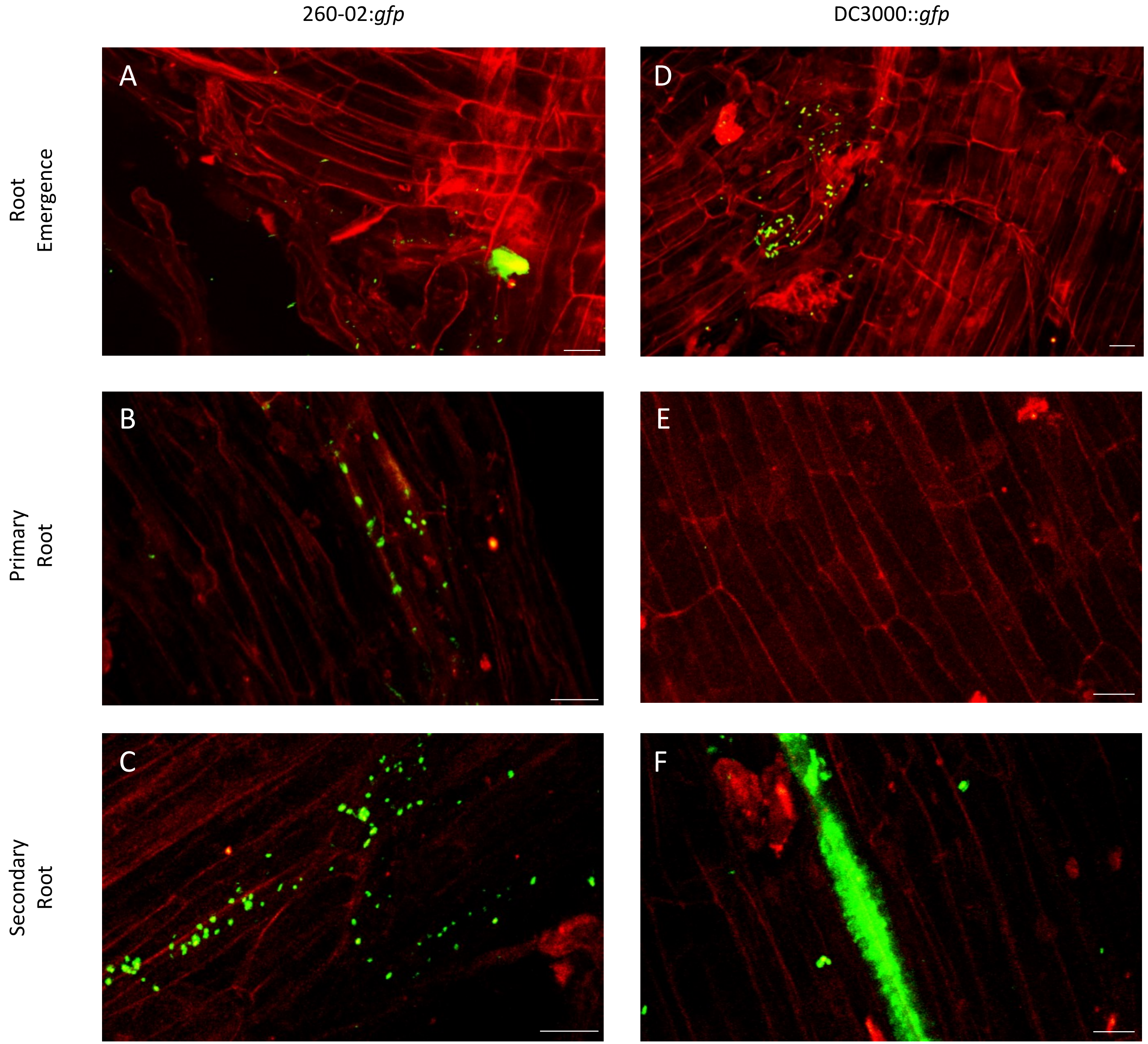

Supplementary file 1B

Confocal microscopy on tomato roots at 3 dpi. The panel shows representative pictures from the microscopy observations. In green is the fluorescence obtained by exciting with a wavelength of 488 nm, which produces fluorescence from the GFP used to tag the bacterial strains, in red that obtained by exciting with a wavelength of 561 nm, which produces autofluorescence from plant tissues and visualizes plant material Pictures from A to C are from plants treated with strain 260-02:*gfp*; pictures from D to F are from the plants treated with strain DC3000::*gfp*. Pictures from A and D portray zones of emergence of secondary roots; B and E portray primary roots of the plants; C and F portray secondary roots.
In each picture, the scale bar reported in the lower right corner represents 20 μm.

Tomato plant roots 6 dpi

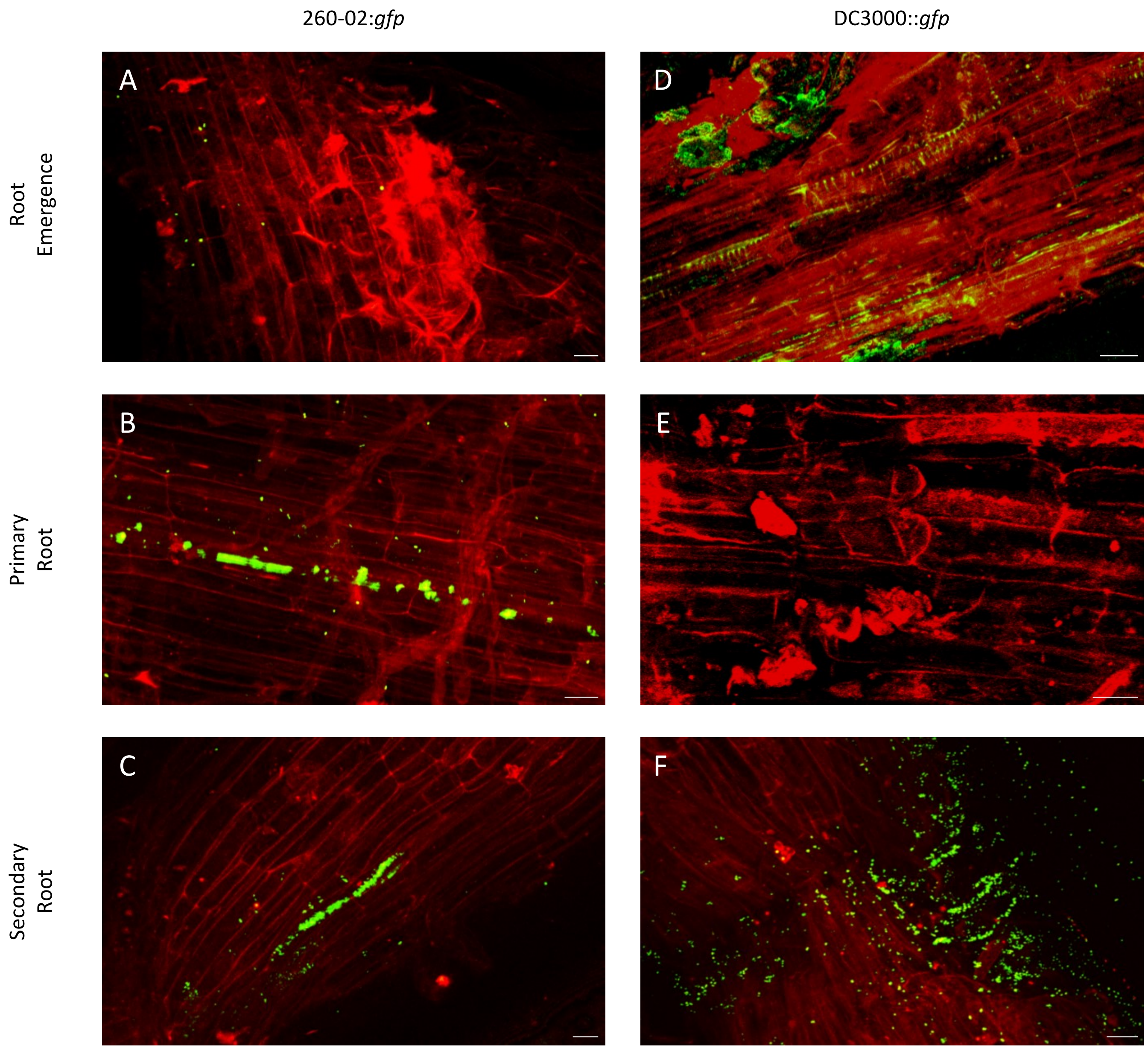

Supplementary file 1C
Confocal microscopy on tomato roots at 6 dpi. The panel shows representative pictures from the microscopy observations In green is the fluorescence obtained by exciting with a wavelength of 488 nm, which produces fluorescence from the GFP used to tag the bacterial strains, in red that obtained by exciting with a wavelength of 561 nm, which produces autofluorescence from plant tissues and visualizes plant material
Pictures from A to C are from plants treated with strain 260-02:*gfp*; pictures from D to F are from the plants treated with strain DC3000::*gfp*. Pictures from A and D portray zones of emergence of secondary roots; B and E portray primary roots of the plants; C and F portray secondary roots.
In each picture, the scale bar reported in the lower right corner represents 20 μm.

Pepper plant roots 6 dpi

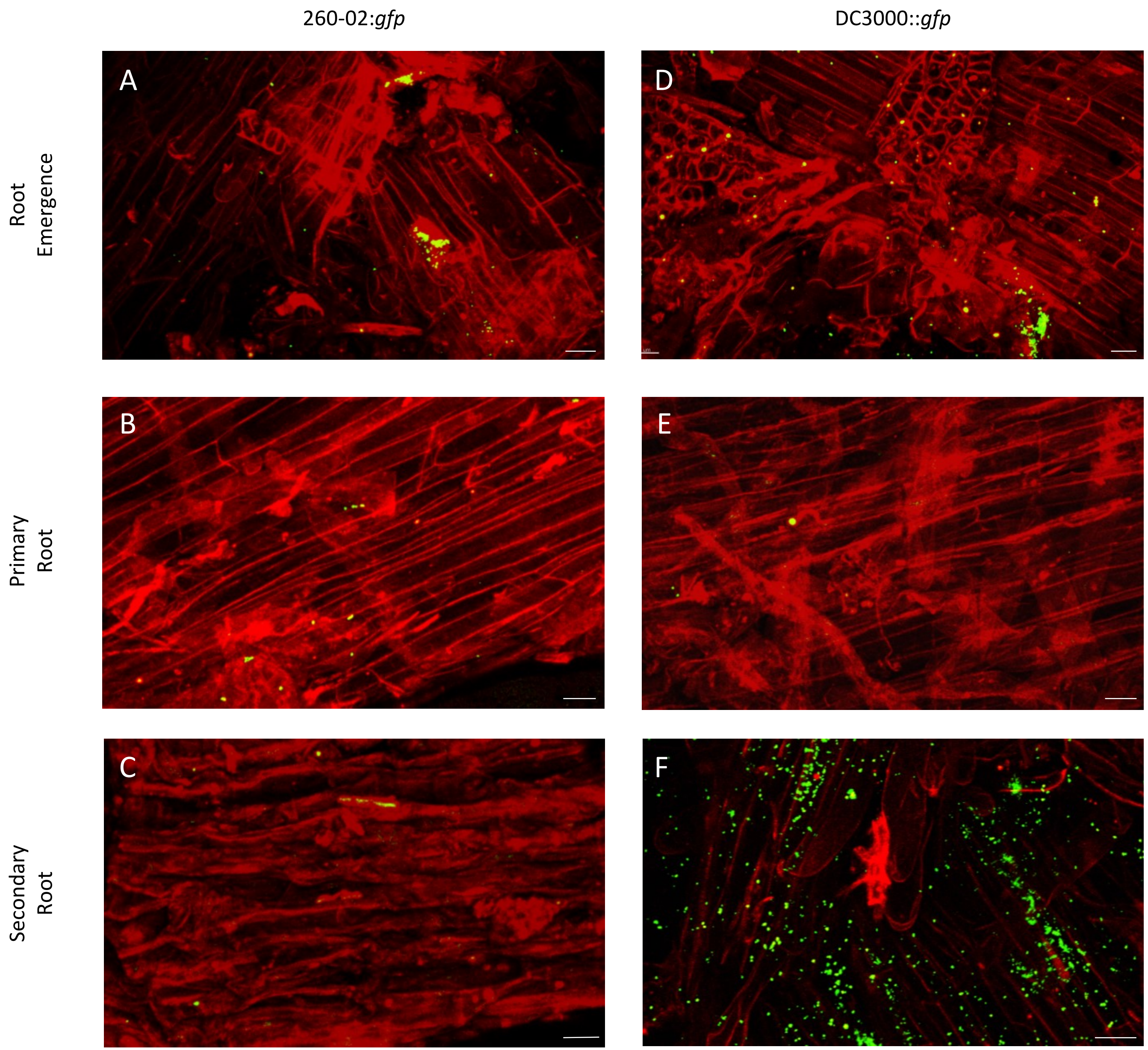

Supplementary file 1D
Confocal microscopy on pepper roots at 6 dpi. The panel shows representative pictures from the microscopy observations. In green is the fluorescence obtained by exciting with a wavelength of 488 nm, which produces fluorescence from the GFP used to tag the bacterial strains, in red that obtained by exciting with a wavelength of 561 nm, which produces autofluorescence from plant tissues and visualizes plant material Pictures from A to C are from plants treated with strain 260-02:*gfp*; pictures from D to F are from the plants treated with strain DC3000::*gfp*. Pictures from A and D portray zones of emergence of secondary roots; B and E portray primary roots of the plants; C and F portray secondary roots.
In each picture, the scale bar reported in the lower right corner represents 20 µm.

Root tip colonization

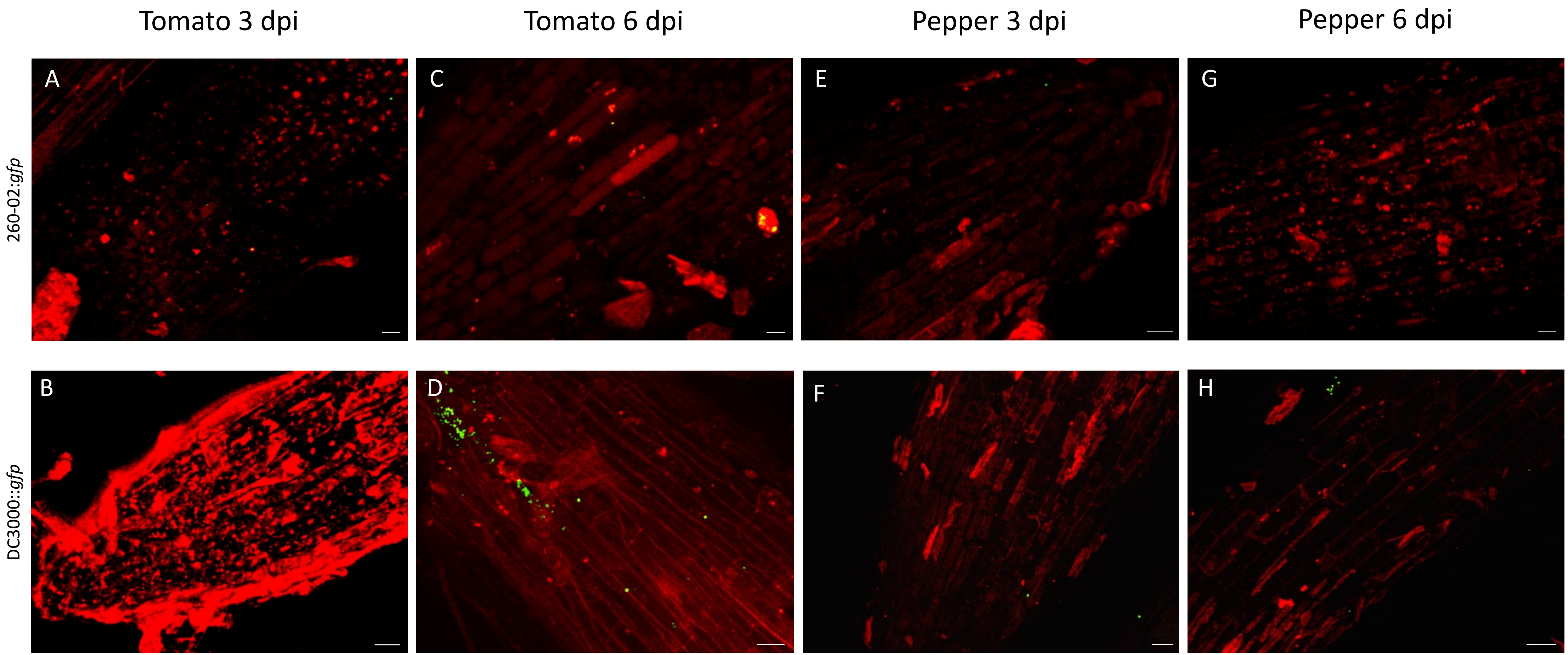

Supplementary file 1E

Confocal microscopy on root tips. The panel shows representative pictures from the microscopy observations. In green is the fluorescence obtained by exciting with a wavelength of 488 nm, which produces fluorescence from the GFP used to tag the bacterial strains, in red that obtained by exciting with a wavelength of 561 nm, which produces autofluorescence from plant tissues and visualizes plant material. Pictures A, C, E and G are from plants treated with strain 260-02:*gfp*; pictures B, D, F, and H are from the plants treated with strain DC3000::*gfp*. Pictures A and B portray root tips of tomato plants at 3 dpi; pictures C and D portray root tips of tomato plants at 6 dpi; pictures E and F portray root tips of pepper plants at 3 dpi; pictures G and H portray root tips of pepper plants at 6 dpi.

In each picture, the scale bar reported in the lower right corner represents 20 µm.

Tomato plant leaves 3 dpi

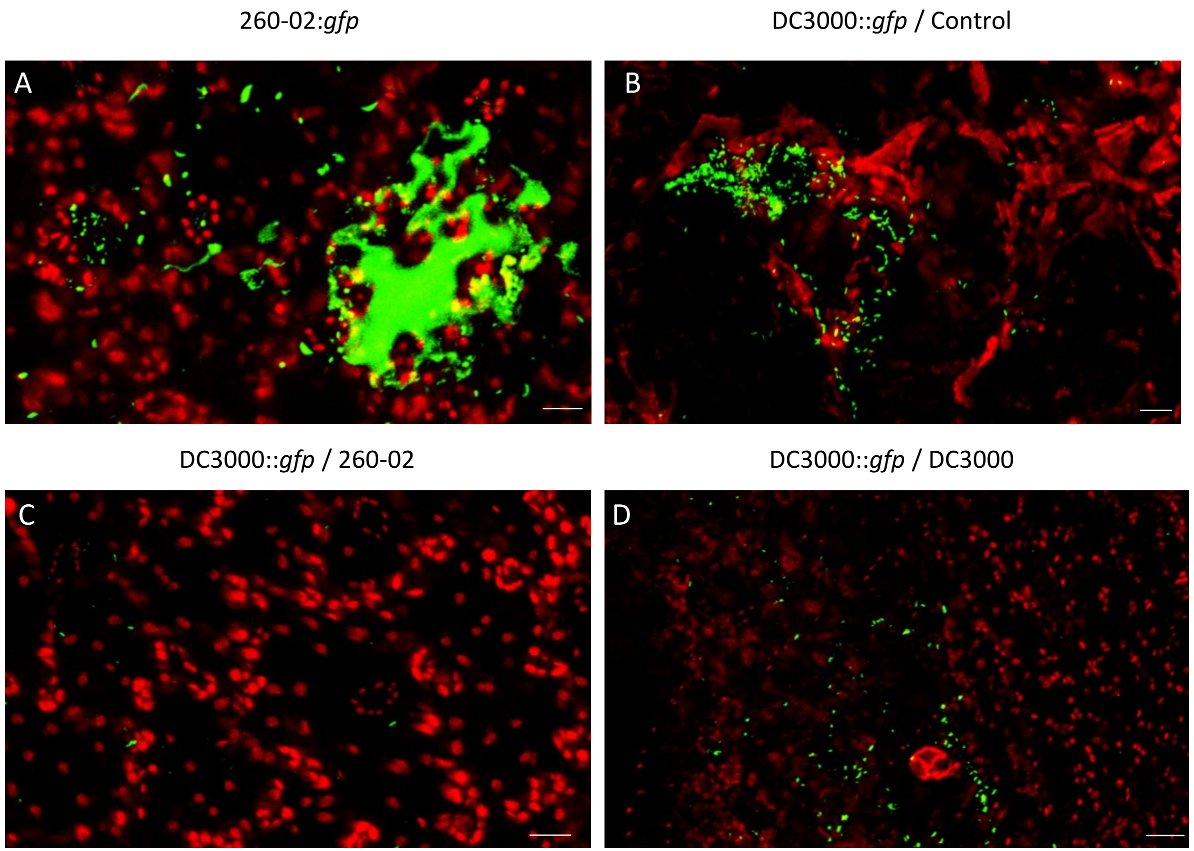

Supplementary file 1F
Confocal microscopy on tomato leaves at 3 dpi. The panel shows representative pictures from the microscopy observations. In green is the fluorescence obtained by exciting with a wavelength of 488 nm, which produces fluorescence from the GFP used to tag the bacterial strains, in red that obtained by exciting with a wavelength of 561 nm, which produces autofluorescence from plant tissues and visualizes plant material.
Picture A is from a plant sprayed with strain 260-02:*gfp*; picture B is from a plant sprayed with strain DC3000::*gfp* without root treatment; picture C is from a plant sprayed with strain DC3000::*gfp* after being treated at the root with strain 260-02; picture D is from a plant sprayed with strain DC3000::*gfp* after being treated at the root with strain DC3000.
In each picture, the scale bar reported in the lower right corner represents 20 µm.

Tomato plant leaves 6 dpi

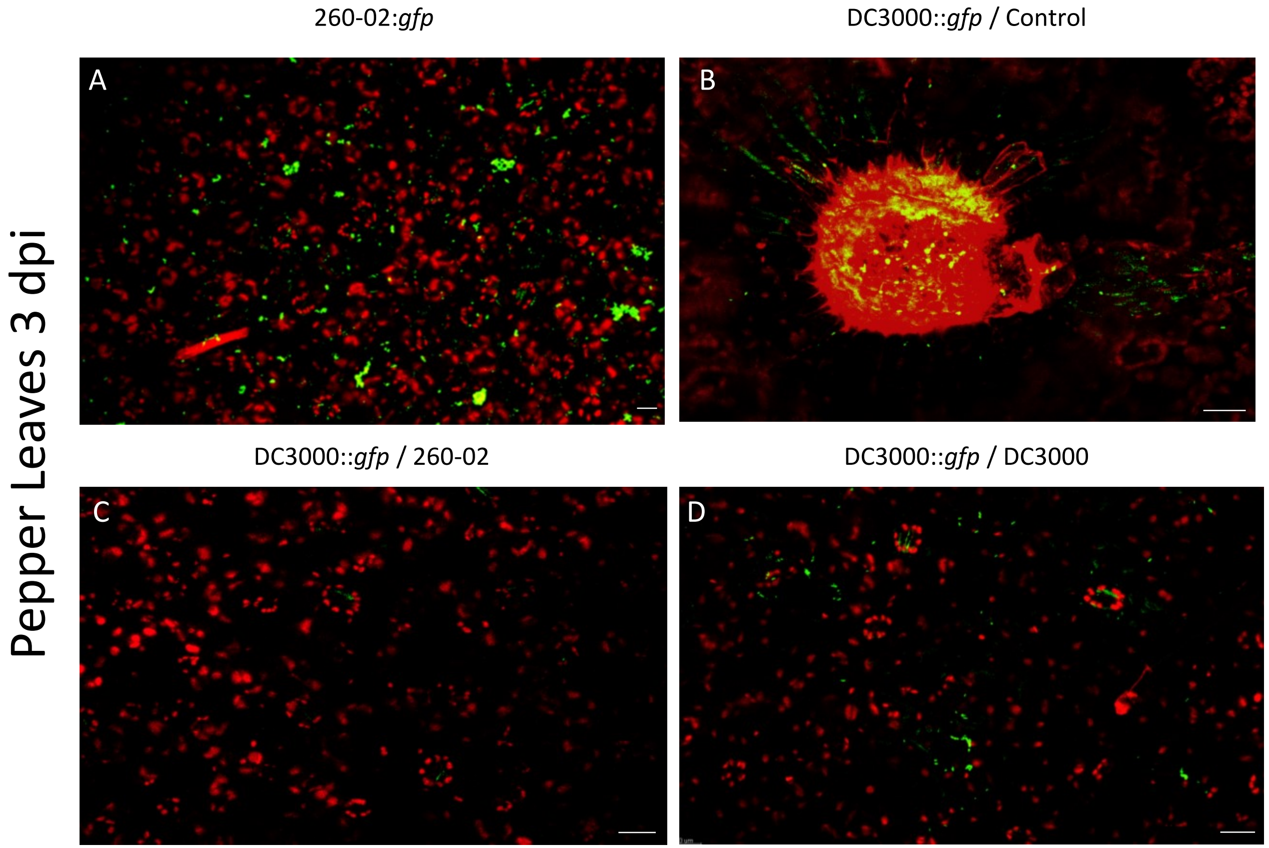

Supplementary file 1G
Confocal microscopy on tomato leaves at 6 dpi. The panel shows representative pictures from the microscopy observations. In green is the fluorescence obtained by exciting with a wavelength of 488 nm, which produces fluorescence from the GFP used to tag the bacterial strains, in red that obtained by exciting with a wavelength of 561 nm, which produces autofluorescence from plant tissues and visualizes plant material.
Picture A is from a plant sprayed with strain 260-02:*gfp*; picture B is from a plant sprayed with strain DC3000::*gfp* without root treatment; picture C is from a plant sprayed with strain DC3000::*gfp* after being treated at the root with strain 260-02; picture D is from a plant sprayed with strain DC3000::*gfp* after being treated at the root with strain DC3000. In each picture, the scale bar reported in the lower right corner represents 20 μm.

Pepper plant leaves 6 dpi

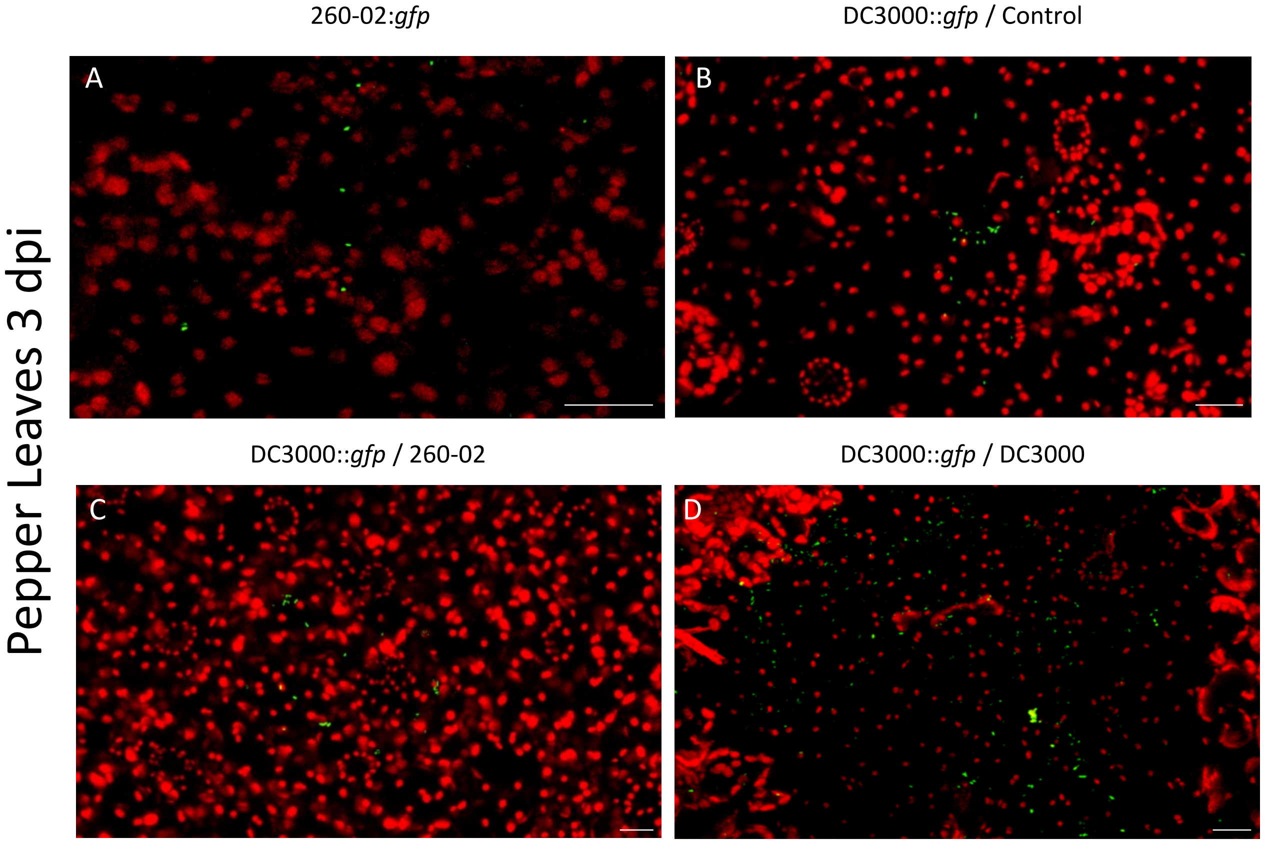

Supplementary file 1H
Confocal microscopy on pepper leaves at 6 dpi. The panel shows representative pictures from the microscopy observations. In green is the fluorescence obtained by exciting with a wavelength of 488 nm, which produces fluorescence from the GFP used to tag the bacterial strains, in red that obtained by exciting with a wavelength of 561 nm, which produces autofluorescence from plant tissues and visualizes plant material.
Picture A is from a plant sprayed with strain 260-02:*gfp*; picture B is from a plant sprayed with strain DC3000::*gfp* without root treatment; picture C is from a plant sprayed with strain DC3000::*gfp* after being treated at the root with strain 260-02; picture D is from a plant sprayed with strain DC3000::*gfp* after being treated at the root with strain DC3000. In each picture, the scale bar reported in the lower right corner represents 20 µm.
